# STAMP: Single-cell transcriptomics analysis and multimodal profiling through imaging

**DOI:** 10.1016/j.cell.2025.05.027

**Published:** 2025-06-17

**Authors:** Emanuele Pitino, Anna Pascual-Reguant, Felipe Segato-Dezem, Kellie Wise, Irepan Salvador-Martinez, Helena Lucia Crowell, Maycon Marção, Max Ruiz, Elise Courtois, William F. Flynn, Santhosh Sivajothi, Emily Soja, Ginevra Caratù, German Atzin Mora-Roldan, B. Kate Dredge, Yutian Liu, Hannah Chasteen, Monika Mohenska, Juan C. Nieto, Raymond K.H. Yip, Ruvimbo D. Mishi, José M. Polo, Mohmed Abdalfttah, Adrienne E. Sullivan, Jasmine T. Plummer, Holger Heyn, Luciano G. Martelotto

**Affiliations:** 1 Centro Nacional de Análisis Genómico (CNAG), Barcelona, Spain; 2 Universitat de Barcelona (UB), Barcelona, Spain; 3 Center for Spatial Omics, St. Jude Children’s Research Hospital, Memphis, TN, USA; 4 Department of Developmental Neurobiology, St. Jude Children’s Research Hospital, Memphis, TN, USA; 5 Adelaide Centre for Epigenetics, School of Biomedicine, the University of Adelaide, Adelaide, SA, Australia; 6 South Australian immunoGENomics Cancer Institute, The University of Adelaide, Adelaide, SA, Australia; 7 The Jackson Laboratory for Genomic Medicine, Farmington, CT, USA; 8 Department of Anatomy & Developmental Biology, Biomedicine Discovery Institute, Monash University, Clayton, VIC, Australia; 9 Department of Cellular and Molecular Biology, St. Jude Children’s Research Hospital, Memphis, TN, USA; 10 The Walter and Eliza Hall Institute of Medical Research, Parkville, VIC, Australia; 11 Spanish National Cancer Research Centre (CNIO), Madrid, Spain; 12 Comprehensive Cancer Center, St. Jude Children’s Research Hospital, Memphis, TN, USA; 13 ICREA, Barcelona, Spain; 14 These authors contributed equally; 15 Lead contact

## Abstract

Single-cell RNA sequencing has revolutionized our understanding of cellular diversity but remains constrained by scalability, high costs, and the destruction of cells during analysis. To overcome these challenges, we developed STAMP (single-cell transcriptomics analysis and multimodal profiling), a highly scalable approach for the profiling of single cells. By leveraging transcriptomics and proteomics imaging platforms, STAMP eliminates sequencing costs, enabling cost-efficient single-cell genomics of millions of cells. Immobilizing (stamping) cells in suspension onto imaging slides, STAMP supports multimodal (RNA, protein, and H&E) profiling, while retaining cellular structure and morphology. We demonstrate STAMP’s versatility by profiling peripheral blood mononuclear cells, cell lines, and stem cells. We highlight the capability of STAMP to identify ultra-rare cell populations, simulate clinical applications, and show its utility for large-scale perturbation studies. In total, we present data for 10,962,092 high-quality cells/nuclei and 6,030,429,954 transcripts. STAMP makes high-resolution cellular profiling more accessible, scalable, and affordable.

## In brief

Single-cell transcriptomics analysis and multimodal profiling (STAMP) by imaging enables single-cell analysis of cells in suspension without the need for sequencing. The markedly reduced costs and flexible experimental designs support the profiling of millions of cells or the large-scale multiplexing of conditions, perturbations, and sample types.

## Graphical Abstract

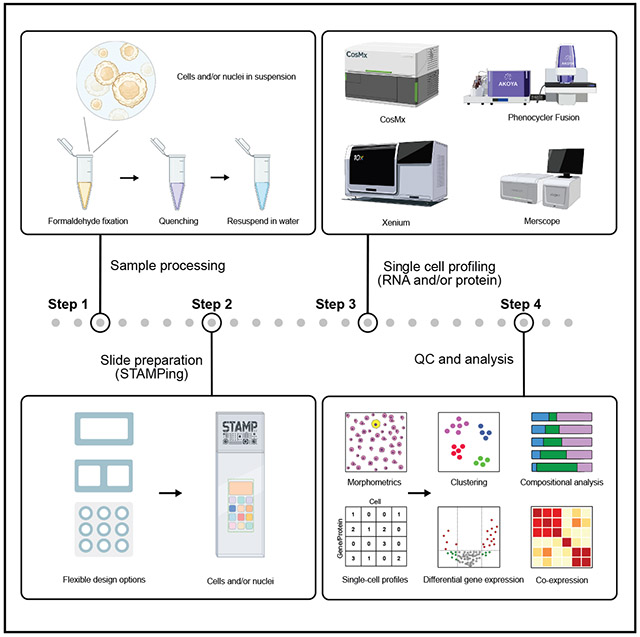

## INTRODUCTION

Over the last decade, single-cell sequencing, particularly single-cell RNA sequencing (scRNA-seq), has revolutionized our understanding of complex tissues and organs by providing high-resolution maps of individual cells. Profiling single cells has offered invaluable insights into cellular composition and states under both steady conditions and dynamic processes, such as differentiation- or disease-associated perturbations.^1^ Innovations in microfluidics and combinatorial indexing have enabled the profiling of increasingly large cell numbers, leading to the generation of organism-wide atlases, including the fly, mouse, and human.^2-4^

Despite these advancements, current single-cell transcriptomic methods face significant limitations. The reliance on sequencing and the requirement to index cells individually in wells or droplets results in high costs that scale linearly with the number of cells. Additional challenges include the random sampling of cellular transcripts, which introduces bias and leads to an overrepresentation of highly abundant transcripts at the expense of lowly expressed genes, such as transcription factors. Single-cell sequencing methods also suffer from inherent inefficiencies. Droplet-based microfluidics are prone to cell damage, inefficient encapsulation, and droplet instability, causing significant sample loss. Plate-based combinatorial indexing methods, meanwhile, exhibit limited cell capture, cross-contamination, and inefficient indexing. Moreover, factors such as cell size, fragility, and RNA content can further affect the capture efficiency, resulting in the under-representation or complete omission of certain cell populations.^5^ Consequently, current single-cell datasets may not accurately reflect the true cellular composition and complexity of a sample.

Conventional scRNA-seq workflows, whether used alone or in combination with multimodal^6^ or multiplexing^7^ strategies, have relatively low throughput, typically processing thousands of cells per experiment. Existing methods also struggle at both ultra-low (hundreds of cells) and ultra-high (millions of cells) scales, with the latter constrained by the high costs for library preparation and sequencing^8^ and high input material requirements.^5,9^ However, scaling single-cell profiling is essential for studying rare cell populations and for capturing the full cell complexity and plasticity in health and disease. Additionally, sequencing-based methods destroy cells during molecule capture, limiting the ability to combine molecular profiles with cell structure and morphology (e.g., shape and size) or functional attributes (e.g., metabolic activity).^10^

To address these limitations, we hypothesized that imaging-based transcriptomics and proteomics readouts of single cells could significantly scale-up cell numbers at substantially reduced costs, while retaining the advantages of single-cell feature detection. Recent advancements in spatial transcriptomic imaging assays have significantly expanded gene panel designs, moving toward transcriptome-wide scales with the potential to match and surpass the capabilities of current scRNA-seq methods.^11,12^ Traditionally, spatial transcriptomics and proteomics have been applied to tissue profiling, mapping composition and architecture of complex samples such as organs or tumors.^12-15^ In this study, we transformed four imaging platforms, the Xenium Analyzer (10× Genomics), the CosMx Spatial Molecular Imager (SMI, Nanostring Technologies/Bruker), the MERSCOPE (Vizgen) and the PhenoCyler Fusion (Akoya Biosciences), into scalable and flexible single-cell profiling tools. We termed this approach single-cell transcriptomics analysis and multimodal profiling (STAMP) through imaging. The adaptation of single-molecule imaging enables the analysis of hundreds to millions of individual cells with flexibility and at scale. STAMP designs support single-modal (RNA or protein) or multimodal (RNA, protein, and H&E) profiling in single- or multi-sample configurations. We demonstrated its utility across diverse sample types and experimental scenarios and compared its quality control metrics to classical single-cell analysis assays.

## RESULTS

### Sequencing-free single-cell genomics through imaging

To address current limitations in single-cell genomics, we developed STAMP, a flexible and scalable approach for cost-efficient, massive-parallel single-cell profiling. STAMP enables the profiling of single cells on slides integrated with state-of-the-art transcriptomic and protein imaging platforms, such as the Xenium Analyzer, the CosMx SMI, and the Phenocyler Fusion. The STAMP workflow begins with the fixation and permeabilization of cells in suspension, followed by anchoring (stamping) cells onto instrument-compatible glass slides to form monolayers. Flexibility is achieved through the adaptable format of stamping areas, allowing versatile multi-sample profiling. After stamping, the cells are hybridized with gene set probes or oligo-conjugated antibodies, followed by cyclic decoding through imaging, which can accommodate any gene or protein panel size and design. This versatility allows STAMP to be used for targeted or data-driven, hypothesis-free strategies to explore the complexity of hundreds to millions of cells in a single experiment.

**Figure 1.**
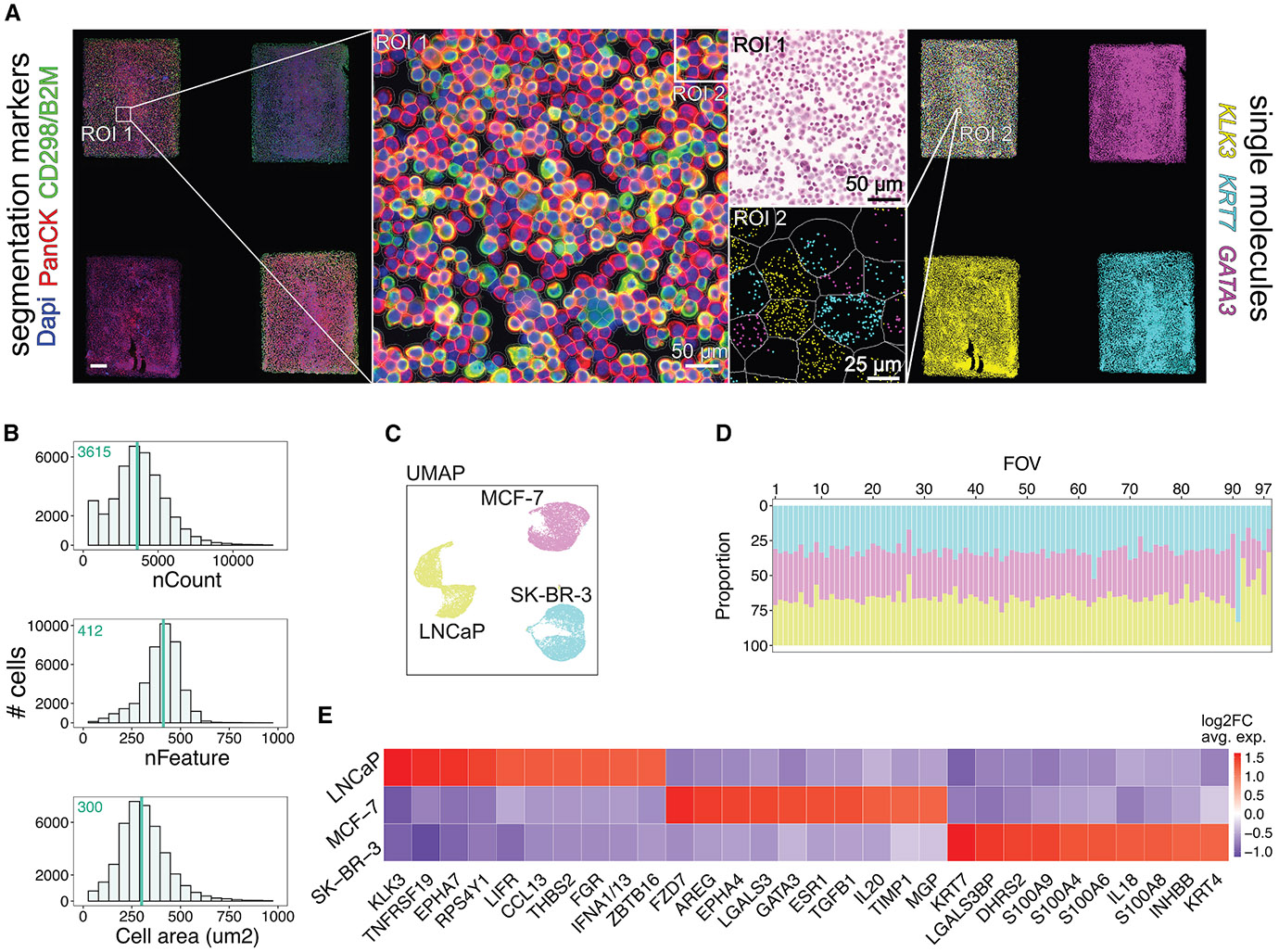
Sequencing-free single-cell genomics through imaging (A) Immunofluorescence (IF) image of STAMP-C highlighting DAPI staining (blue), CD298/B2M (red), and pan-cytokeratin (PanCK, green) across the four sub-STAMPs. Marker genes specific to cell lines are highlighted: *KLK3* for LNCaP (yellow), *GATA3* for MCF-7 (magenta), and *KRT7* for SK-BR-3 (cyan). Enlarged region of interest (ROI1) with post-STAMP hematoxylin and eosin staining. In ROI2, each dot represents a transcript and white lines indicate segmented cell borders (scale bar, 750 μm unless otherwise noted). (B) Quality metrics for the mixed sub-STAMP containing equal proportions of MCF-7, LNCaP, and SK-BR-3 cell lines (median values of transcript counts, features, and cell areas prior to filtering). (C) UMAP of the mixed sub-STAMP, colored by InSituType unsupervised clustering. (D) Composition of the 99 fields of view (FOVs) in the mixed sub-STAMP (as in C). (E) Heatmap displaying the top 10 marker genes for each cluster identified in (C) normalized by feature. Related to Figure S1.

To evaluate STAMP’s suitability and specificity for imaging-based transcriptomics, we initially profiled three cancer cell lines (LNCaP, MCF-7, and SK-BR-3) using the CosMx SMI platform (STAMP-C) with the 1000-plex Human Universal Cell Characterization RNA Panel. The multi-sample slide array contained four sub-STAMPs, each containing ~35,000 cells: three with individual cancer cell lines and one with a 1:1:1 pooled mixture (Figures 1A and S1A). For imaging, we selected contiguous fields of view (FOVs) to comprehensively scan each sub-STAMP. We also obtained cell staining information for DAPI, pancytokeratin (PCK), and pan-membrane markers (B2M/CD298) used for cell segmentation (see STAR Methods; Figure 1A). We initially confirmed a uniform distribution of transcripts, genes, and cell areas across each sub-STAMP pointing to homogeneous cell stamping (Figures S1B-S1D). We then interrogated the pooled mixture to distinguish different cell types in a spatially mixed environment. On average, we obtained 11,103 cells per cell line, with a median of 3,637 transcripts and 413 genes per cell (Figure 1B). Stringent quality control steps included removing segmentation artifacts, filtering out low transcript and gene counts (<2.5 median absolute deviations, MAD), and excluding high counts and large cell areas (>2.5 MAD). Cells near the FOV borders (<30 pixels) were also excluded due to reduced counts and features (Figure S1E). Overall, 4.63% of cells were removed from the analysis (Figure S1F).

Using the InSituType (IST)^16^ algorithm for unsupervised clustering, we identified three distinct clusters with unique transcriptional profiles and an average of 33.33% (range: 32.6%–33.4%) of each cell line per FOV (Figures 1C-1E). To validate the clustering results of the pooled mixture, IST was applied to the sub-STAMPs of the individual cell lines, which also separated into three distinct clusters with transcriptional profiles matching those found in the mixture (Figures S1G and S1H). The gene signatures of both pooled and individual cell lines were correlated with data from a scRNA-seq reference dataset generated using the Single-Cell Gene Expression Flex assay (10 × Genomics) on the same suspension of fixed cancer cells (Figure S1I).

### Sensitive capture of low-input cell numbers

**Figure 2.**
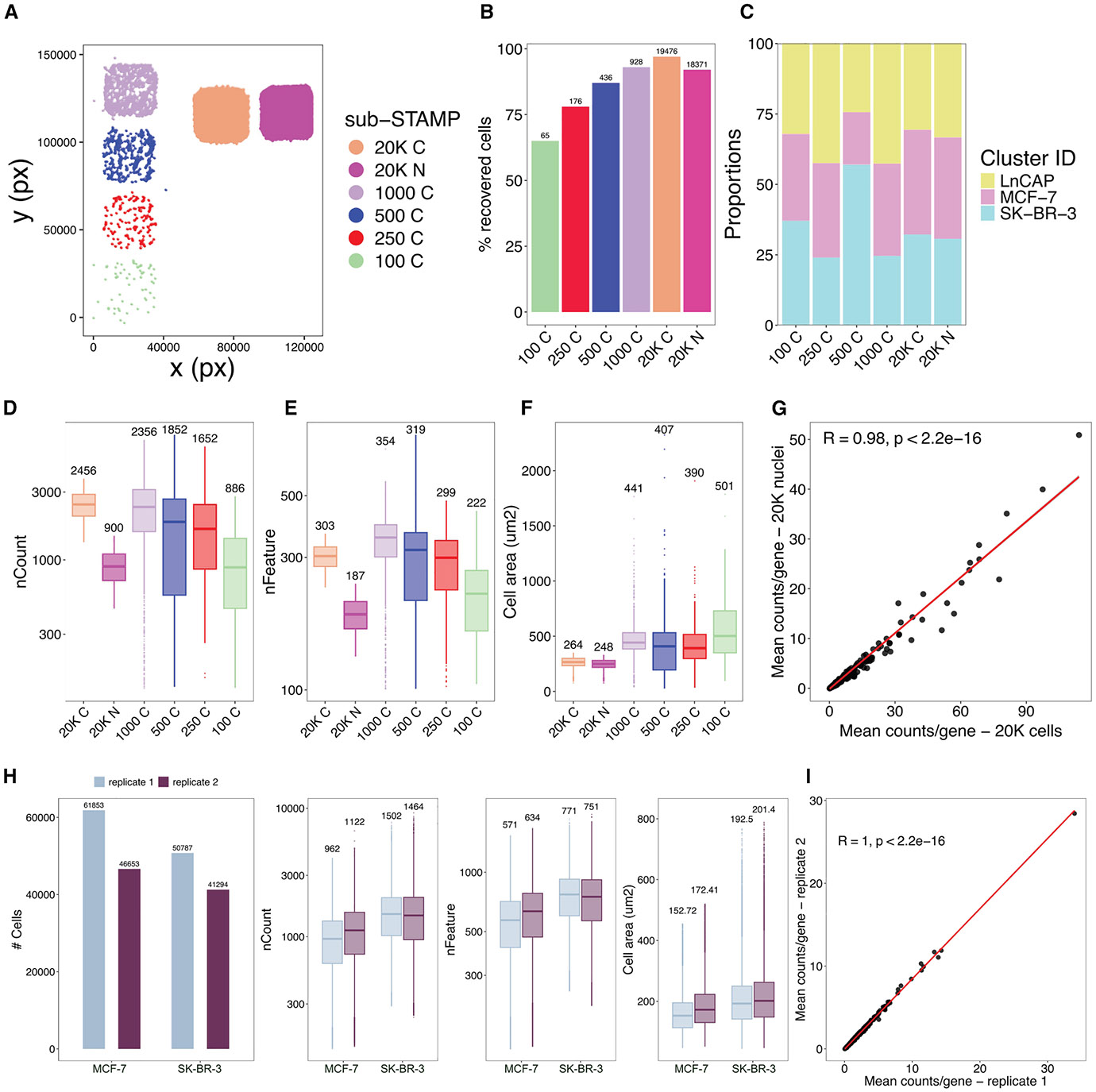
Sensitive capture of low-input cell numbers (A) STAMP-C with 6 sub-STAMPs with low cell seeding (sub-STAMPs: 100, 250, 500, 1,000, and 20,000 cells and 20,000 nuclei; C: cells, N: nuclei). Each dot is a cell detected by imaging (px: pixels). (B) Percentage of cells retrieved from input across sub-STAMPS. Labels indicate absolute cell counts. (C) Proportions of tumor cell lines across sub-STAMPs. (D–F) Boxplot displaying the number of counts (D), features (E), and cell area (F) for each sub-STAMP. (G) Pearson correlation of raw counts averaged by cell number between cells and nuclei. Each dot is a gene (red line shows the fitted linear regression). *p* < 0.05. (H) Number of detected cells, transcripts, genes, and cell area for each cell line in each STAMP-X (replicate 1 and 2). (I) Pearson correlation of raw counts averaged by cell number between MCF-7 and SK-BR-3 replicates. *p* < 0.05.

To explore the scalability to ultra-low cell densities, we stamped cancer cell line mixtures (1:1:1 ratio of MCF-7, SK-BR-3, and LNCaP) into four sub-STAMPs with varying cell counts of approximately 100, 250, 500, and 1,000 cells (Figure 2A). This setup allowed us to assess the performance at low cell numbers, which are challenging to capture with current droplet microfluidics or combinatorial indexing scRNA-seq methods. Additionally, we profiled ~20,000 cells and ~20,000 nuclei of the cell line mixture separately in two sub-STAMPs, enabling a direct side-by-side comparison of high-RNA (whole cell) and low-RNA (nuclear) content samples within a multiplexed experimental setup. The mean recovery rate of high-quality cells was 85.33% (range: 65%–97%) from the input material (Figure 2B), with an average mix of 32.5% for each cell line per sub-STAMP (range: 18.6%–56.9%, Figure 2C). The median gene counts per cell were consistent across sub-STAMPs representing higher loadings, with reduced genes detected in the lowest sub-STAMP (100 cells; Figure 2D). The number of detected features per cell followed similar trends, indicating suboptimal performance when profiling extremely low cell densities (Figure 2E). Cell areas remained stable in less densely seeded sub-STAMPs but halved in those with higher confluence, likely due to reduced cell expansion during segmentation (Figure 2F). Expectedly, nuclei had fewer counts and features than intact cells, yet gene expression levels were highly correlated across cells and nuclei (Figures 2D, 2E, and 2G).

We next tested the reproducibility of our approach by splitting MCF-7 and SK-BR-3 cell suspensions into two aliquots, imaged in two slides as technical replicates (scanned in the same Xenium run using the Xenium Prime 5k Human Pan Tissue and Pathways panel, STAMP-X). Although recovered cell numbers slightly varied, both replicates showed a highly consistent number of transcripts and genes detected, as well as equal cell areas (Figure 2H). Accordingly, gene expression levels correlated highly across replicates (Figure 2I).

### Sample multiplexing across platforms

**Figure 3.**
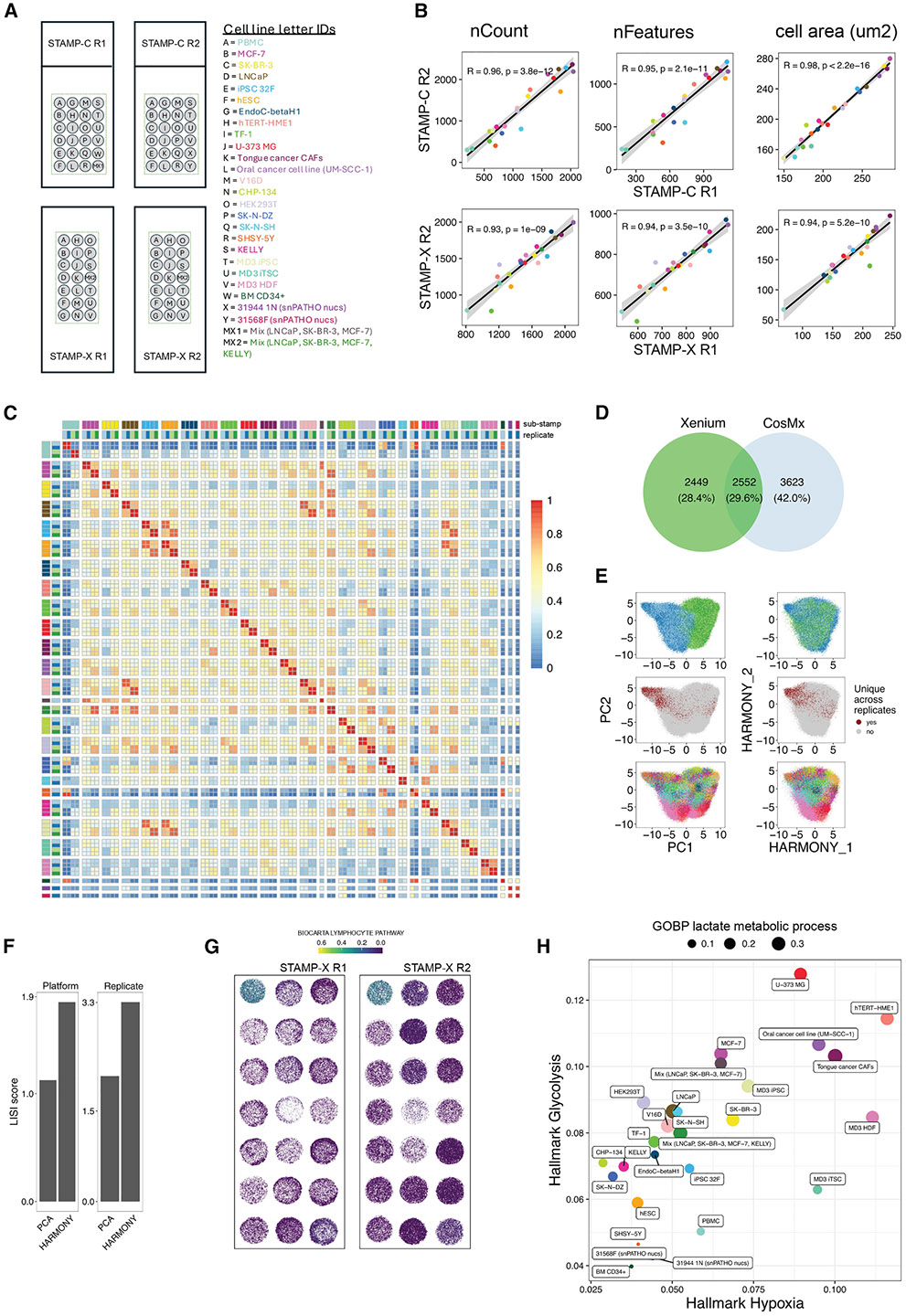
Sample multiplexing across platforms (A) Layout of multiplexed conditions profiled with the STAMP-C (top) and STAMP-X (bottom), each with replicates. (B) Pearson correlations comparing the number of counts, features, and cell area across replicates for STAMP-C (top) and STAMP-X (bottom). *p* < 0.05. (C) Gene-wise aggregated counts showing high Pearson correlations across samples and replicates. (D) Overlap of genes between the Xenium Prime 5K Human Pan Tissue & Pathways Panel and the CosMx Human 6K Discovery Panel, demonstrating complementary profiling capabilities. (E) Principal-component analysis (PCA) plots of unintegrated data (left) and integrated data (right), colored by technology (top), replicate uniqueness (middle), and sample identity (bottom). (F) Local inverse Simpson’s index (LISI) scores calculated by technology (left) and replicate (right), illustrating improved data integration performance. (G) AUCell scores for the Biocarta lymphocyte pathway across Xenium replicates. (H) Scatterplot of AUCell scores for Hallmark Hypoxia (*x* axis) and Hallmark Glycolysis (*y* axis). Each point represents a sample (point size: AUCell GOBP lactate metabolic process).

To evaluate the multiplexing capabilities of STAMP, we prepared 27 distinct samples, comprising PBMCs, stem cells, cancer cell lines, cancer-associated fibroblasts (CAFs), cancer cell lines (see STAR Methods and Table S1), nuclei samples extracted from prostate cancer FFPE tissues, and mixed cell populations. The samples were stamped onto two replicate slides for each assay, STAMP-C and STAMP-X, with 21 sub-STAMPs shared between the assays. This setup ensured consistency and reproducibility, while enabling a comprehensive evaluation of the assays’ multiplexing capabilities (Figure 3A). We selected the largest gene panels available for each platform to match the cell type diversity represented by the sub-STAMPs. Specifically, we used the Xenium Prime 5k Human Pan Tissue and Pathways Panel and the CosMx Human 6K Discovery Panel.

Gene and transcript number per cell, as well as cell areas, were highly correlated across replicates, once again underscoring the robustness of the assay (Figure 3B). Furthermore, experimental conditions at both the upper and lower ends of the gene, molecule count, and area distributions were consistent across platforms. Aggregating genes across all samples and replicates revealed high correlations, not only within replicates but also across platforms in 18 out of 21 conditions (Figure 3C). The suboptimal correlation observed for CHP-134, SK-N-DZ, and KELLY cell lines may be explained by the low overlap between the two panels (29,6%, Figure 3D). High correlations were also detected across biologically similar samples. For instance, iPSC 32F, hESC, and MD3iPSC clustered together, as did LNCaP and V16D (a castration-resistant prostate cancer cell line derived from LNCaP cells). Similar trends were evident for individual cancer cell lines LNCaP, SK-BR-3, and MCF-7 compared with their pooled mixtures (MX1 and MX2).

Given the high correlation of STAMP across platforms, we performed principal-components analysis (PCA) on all datasets together. The batch effect introduced by the panels and platforms, captured by PC1 was successfully removed using Harmony integration^17^ (Figure 3E). This integration also improved the local inverse Simpson’s index (LISI score), reflecting consistency across both platforms (technical) and replicate batches (Figure 3F). Next, we analyzed curated pathways from the MSigDB database (https://www.gsea-msigdb.org/gsea/index.jsp) on the sub-STAMPs using AUCell.^18^ As an example, we identified a specific enrichment in the Biocarta Lymphocyte Pathway on the stamped PBMCs, which was consistent across replicates (Figure 3G). Hallmark Glycolysis and Hypoxia pathways and GOBP Lactate metabolic processes were similarly enriched in the cancer cell lines but showed low scores in bone marrow progenitor cells and prostate cancer FFPE nuclei (Figure 3H). These findings align with the enhanced lactate production under hypoxic conditions due to increased glycolytic rates, commonly observed as a metabolic adaptation in cancer cells^19^ and differentiated cell lines in *in vitro* cultures.^20^

We further tested whether STAMP could be applied to the MERSCOPE platform (Vizgen) and applied the PanNeuro Cell Type Panel (500 genes) across 12 of the previously analyzed cell lines (STAMP-M; Figure S2A; Table S1). In total, we profiled 285,445 cells with a median of 194 transcripts (SD = 318.7) and 102 unique genes per cell (SD = 70.05, Figures S2B-S2E). STAMP-M profiling returned similar cell area sizes as observed for the previous STAMP assays, with the majority of cells being of high quality (251,199 cells, 88%). A one-vs-all differential expression analysis identified specific gene markers for each cell line, confirming the applicability of STAMP-M for cellular phenotyping (Figure S2G). These results show that STAMP enables high-level multiplexing on a single slide, facilitating the simultaneous analysis of multiple samples across various imaging platforms.

### Immuno-phenotyping of millions of circulating blood cells

**Figure 4.**
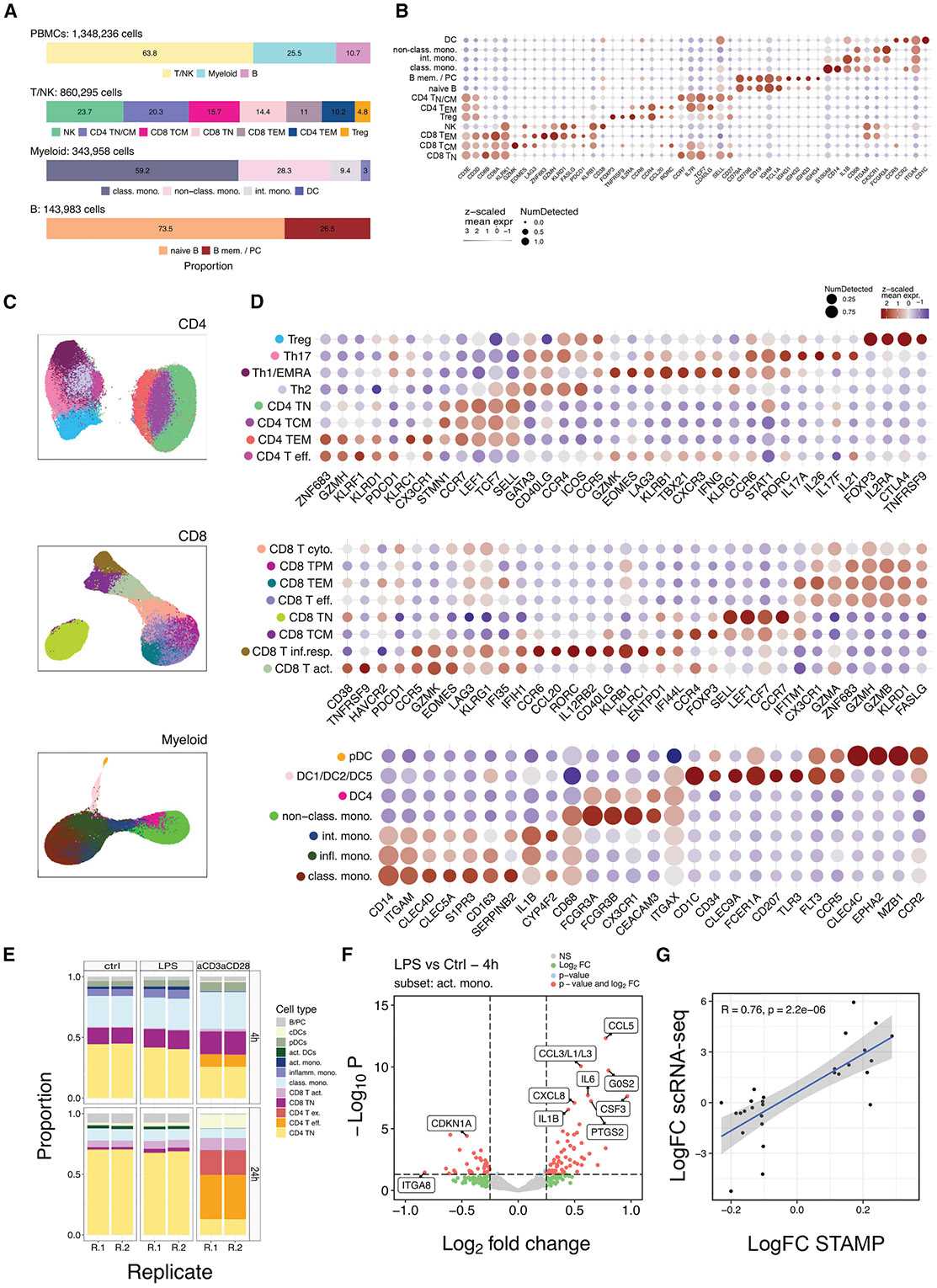
Immuno-phenotyping of millions of circulating blood cells (A) Cell numbers and proportions for the full PBMC dataset by immune lineage. (B) Dot plot displaying cell-type-defining marker genes normalized by feature. (C) UMAP visualizations of T cell and myeloid compartments (cells colored by cluster). (D) Dot plot showing normalized expression of cell-type-specific marker genes for clusters identified in (C). (DC, dendritic cells; pDC, plasmacytoid dendritic cells; non-class. Mono, non-classical monocytes; int. Mono, intermediate monocytes; infl. mono, inflammatory monocytes; class. Mono, classical monocytes; TN, naive T cells; TCM, central memory T cells; TEM, effector memory T cells; TPM, peripheral memory T cells; T eff, effector T cells; T act, activated T cells; T cyto, cytotoxic T cells; T inf. resp, interferon responder T cells. (E) Cell type proportions of PBMCs cultured under control conditions (left), LPS stimulation (middle), or anti-CD3/CD28 stimulation (right) at 4 h (top) and 24 h (bottom). (F) Volcano plot of differentially expressed genes in activated monocytes, comparing LPS versus control (4 h). (G) Pearson correlation on LogFC of genes differentially expressed in both STAMP (x axis) and Flex scRNA-seq (y axis). Related to Figures S3 and S4. *p* < 0.05.

We then expanded the protocol to stamp millions of cells per experiment. A high-density STAMP containing ~1.7 million PBMCs was generated, on which we applied the Xenium Immuno-Oncology panel (380 target genes) and imaged a median of 83 transcripts per cell (range: 27–259) and 49 genes per cell (range: 24–103). After quality control to remove segmentation errors and cells with extreme counts and features (Figure S3A), 88,53% of high-quality cells were retained. Subsequently, we applied conventional single-cell analyses, including dimensionality reduction and PCA (see STAR Methods). Principal components (PCs) 1–3 effectively resolved the three main immune lineages, namely Myeloid, T, and B cells, based on cell lineage marker genes (Figures S3B and S3C). As expected, the myeloid compartment displayed a higher number of genes and transcripts and larger cell areas (Figure S3D). Further clustering of each immune lineage identified all major PBMC cell types and states at expected frequencies, defined by cell state markers (Figures 4A and 4B). In total, 13 immune clusters representing the main PBMC populations were identified.

To test STAMP’s power for high-resolution immuno-phenotyping, we stamped an additional 750,000 cells using a larger probe panel (Xenium Prime 5K Human Pan Tissue and Pathways panel). This analysis generated a high-resolution map of 31 immune cell states in circulation, providing the foundation for large-scale atlasing projects across dimensions such as time (e.g., age) or genetics (e.g., ethnic background; Figures 4C and 4D and Figure S3E). Notably, eight CD4^+^ T cell subsets were identified, ranging from naive to effector and memory populations, including Th1, Th2, and Th17 functional cell types that represent the three arms of adaptive immune responses. Similarly, eight subclusters within the CD8^+^ T cell pool were annotated, ranging from naive to central/effector memory populations and different types of effector populations, including interferon-responding CD8 T cells. Within the circulating NK cell pool, we distinguished CD56^dim^CD16^bright^ from CD56^bright^CD16^dim^ NK cells, which exhibit divergent antibody-dependent cellular cytotoxicity and migratory properties, along with a small proportion of proliferating *EOMES*- and *CD34*-expressing cells, likely representing NK progenitors. Ig-related genes and other markers relevant for B cell phenotyping were missing in the panel, which limited a deeper annotation of B cell differentiation states. However, the myeloid compartment revealed well-defined monocyte subsets (classical, intermediate, and non-classical monocytes) and enabled detailed characterization of DCs.^21^

Next, we benchmarked the sensitivity of transcript and gene detection of STAMP-X (Xenium Prime 5K Human Pan Tissue and Pathways panel) against single-cell sequencing approaches (Figure S4A). Here, STAMP-X showed comparable detection levels as the commonly used 3′, 5′, and Flex single-cell assays (10× Genomics) when analyzing the 4,808 genes overlapping across assays. STAMP-X showed lower sensitivity when analyzing all genes, in line with the gene panel design limitations, compared with the transcriptome-wide coverage of sequencing-based assays. However, the STAMP performance even exceeded the single-cell assays after normalizing by the total number of genes (targeted/captured, Figure S4A), pointing to a high molecule detection efficiency. Finally, STAMP was equal or superior to single-cell assays at detecting lineage-defining marker genes, including transcription factors and surface markers (Figure S4B).

### Multiplexing of immune cell perturbation experiments

We next evaluated the suitability of STAMP for multiplexed perturbation studies, such as the responses to classic immune activators (anti-CD3/CD28 for adaptive and LPS for innate immunity). Therefore, PBMCs were cultured with either media alone (control), LPS, or CD3/CD28 beads, harvested at 4 and 24 h (Figure S3F). Using the CosMx 1000-plex Human Universal Cell Characterization RNA Panel, a median of 320 transcripts (range: 33–1,630) and 183 genes (range: 33–55) were detected per cell, with a median cell area of 78 μm^2^ (Figure S3G). After filtering out low-quality cells (18%), unsupervised clustering with InSituType (IST) identified 12 distinct immune cell clusters (Figure S3G and S3H). In line with the results obtained with the Xenium Immuno-Oncology panel, we captured immune cells from B, T, and myeloid lineages at expected proportions (4%, 68%, and 28%, respectively). Anti-CD3/CD28 stimulation progressively decreased naive CD4^+^ T cells and increased effector and exhausted CD4^+^ T cells at the 4 and 24 h time points (Figure 4E). At 24 h, naive CD8^+^ T cells also decreased markedly, while activated CD8^+^ T cells increased in number. The T cell activation also altered myeloid cell proportions, with classical monocytes and plasmacytoid DCs (pDCs) increasing at 4 h, while inflammatory monocytes decreased. At 24 h, conventional DCs expanded and activated DCs declined relative to the controls. LPS stimulation caused subtler compositional changes, consistent with previous reports.^22^ LPS is known to activate monocytes through the cell surface Toll-like receptor 4 (TLR4) complex,^23^ and, accordingly, activated monocytes showed the highest TLR4 expression levels among the myeloid cell subtypes (Figure S3I). Overall, 39 genes were differentially expressed in activated monocytes (30 up- and 9 downregulated; FDR < 0.05 and log_2_FC > 0.25) upon LPS treatment (4 h), among them were pro-inflammatory cytokine and chemokine genes (e.g., *IL1B, IL6, CCL3, CCL5*, and *CXCL8*) and other inflammation-associated transcripts (e.g., *PTGS2, STAT4*, and *CSF3*; Figure 4F). By 24 h, the upregulation of *DUSP1* and *B2M* and the downregulation of *IL1B* in activated monocytes suggested a transition from early inflammatory responses to regulatory feedback mechanisms to limit inflammation (Figure S3J). We next explored cell-to-cell communication upon LPS activation using CellChat^24^ (Figure S3K). We focused on the CXC pathway, as one of the major secreted signaling pathways induced under inflammatory conditions and controlling immune responses through the migration of leukocytes.^25^ In line with the induced TLR4-mediated activation of monocytes, we detected strong signaling between inflammatory and activated monocytes and CD4 effector T cells. Here, the CXCL8/CXCR1 receptor-ligand pair was the major contributor to the communication between these cell types, which also expressed the highest levels of the chemokine (CXCL8) and its cognate receptor (CXCR1). Finally, we validated the treatment effect identified by STAMP using an independent single-cell-sequencing dataset of LPS-stimulated PBMCs, identifying a significant correlation of the differentially expressed genes between the two assays (Pearson correlation coefficient = 0.76, *p* < 0.01, Figure 4G).

### Profiling cell state dynamics during stem cell differentiation

**Figure 5.**
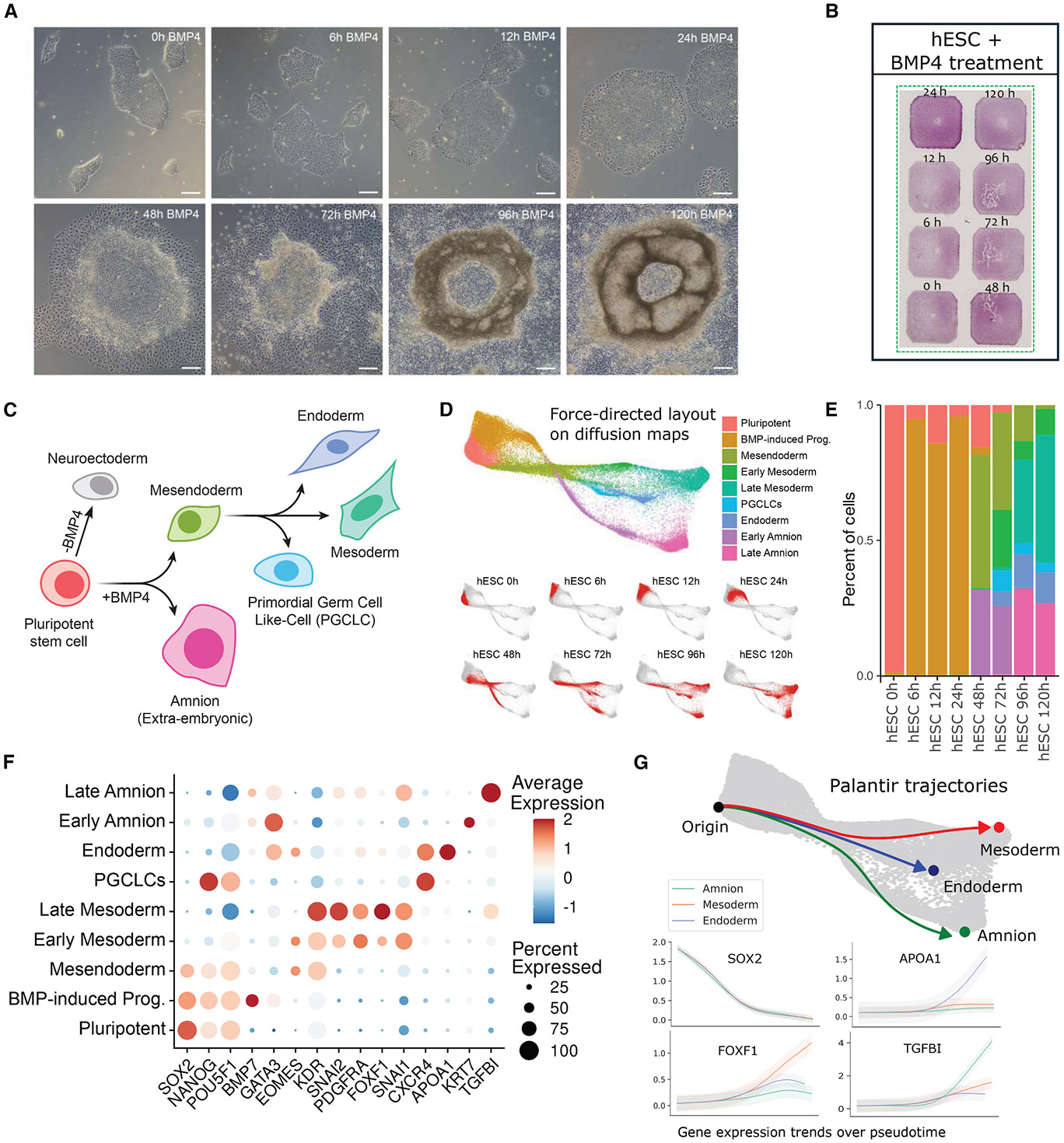
Profiling cell state dynamics during stem cell differentiation (A) Representative images of H1 (WA01) hESCs treated with BMP4 (50 ng/mL) for 0, 6, 12, 24, 48, 72, 96, and 120 h (scale bars: 200 μm). (B) STAMP slide layout of the eight BMP4-treatment time points after H&E-stained post-STAMP-C. (C) Schematic illustrating the expected differentiation trajectories of hESCs following BMP4 induction. (D) A force-directed layout of cells from all time points based on diffusion maps (Palantir). Cells are colored by annotated cell states (top) and displayed separately for each time point (bottom). (E) The proportions of annotated cell states at each time point (as in D). (F) Dot plot highlighting the main marker genes used for cell state annotation. (G) Differentiation trajectories for the amnion, endoderm, and mesoderm branches (Palantir), with expression trends of selected marker genes along the pseudotime trajectories. Related to Figure S5.

We next evaluated the resolution to which cell states emerging from differentiating hESCs in response to BMP4 treatment could be identified, leveraging the multiplexing capabilities of STAMP. Adding BMP4 to cultured hESC colonies triggers spatially resolved signaling cascades, recapitulating those observed in the epiblast during gastrulation. This process results in concentric rings of embryonic germ lineages and the extra-embryonic cell type, amnion.^26-28^ To profile the trajectories of these lineages as they arise, we combined eight BMP4 treatment time points (0–120 h) in a single STAMP-C experiment using the CosMx Human Universal Cell Characterization RNA panel (Figures 5A and 5B). We then applied pseudotime trajectory analysis with Palantir^29^ to model the differentiation path of hESCs in response to BMP4 treatment (Figures 5C-5F). The initial trajectory bifurcation in this system, determined by the presence or absence of BMP pathway activity (Figure 5C), is influenced by the cell position within the colony. Due to the basolateral localization of BMP receptors, cells in the center of large colonies are occluded from BMP4 in the media. However, as this sample contained relatively few large colonies, the majority of BMP4-treated hESCs transitioned from a pluripotent state to an intermediate BMP-induced state as early as 6 h, persisting until 12–24 h after BMP4 exposure. This transition was marked by the rapid upregulation of BMP4 pathway target genes, such as *GATA3*,^30^ followed by a downregulation of pluripotency marker *SOX2*.

By 48 h, a mesendoderm-like state emerged, characterized by the expression of *EOMES* and *KDR*, alongside an early amnion-like state strongly expressing *GATA3*. After 72 h, the mesendoderm-like cells gave rise to early mesoderm-like (*SNAI2* and *PDGFRA*), endoderm-like (*CXCR4* and *APOA1*), and primordial germ-cell-like (PGCLC; *NANOG* and *CXCR4*) cells. By 72 h, early amnion-like cells differentiated into late amnion-like cells expressing *TGFBI*, while the mesoderm branch progressed into a late mesoderm-like state, characterized by upregulation of *DUSP6* and *FOXF1*. Using Palantir, we also computed the gene expression dynamics of key marker genes along the amnion, mesoderm, and endoderm branches, demonstrating the progressive downregulation of pluripotency genes and the upregulation of lineage-defining markers (Figure 5G). Equivalent analyses performed on cells from the same samples, processed with the Single-Cell Gene Expression Flex assay, revealed similar trajectories and a strong positive correlation of the gene expression in both assays across differentiation time points (Figures S5A-S5F). Both trajectories and marker gene dynamics are consistent with previous scRNA-seq studies of hESC-based gastrulation models^26^ and a gastrulating human embryo.^31^

Induced Pluripotent Stem Cells (iPSCs) are pluripotent cells generated from differentiated donor cells, e.g., skin fibroblasts, through cellular reprogramming. Subsequently, these can be differentiated into various cell types and represent an invaluable tool to model human development and genetic diseases.^32,33^ Hence, iPSCs have been used to drive discovery in applications such as disease modeling,^34,35^ drug screening and toxicity testing,^36,37^ regenerative medicine,^38,39^ immune research,^40^ and studying lineage specification. Given these extensive application areas, applying STAMP to iPSCs enlarges the molecular profiling toolbox and significantly broadens its utility across multiple research fields. To explore such potential, we differentiated iPSCs into (neuro)ectoderm and mesodermal lineages and performed scRNA-seq using the Single-Cell Gene Expression Flex assay alongside STAMP-C, using the CosMx 1000-plex Human Universal Cell Characterization RNA Panel. Dimensionality reduction and PCA showed the first two PCs to resolve undifferentiated parental cells, ectoderm, and mesoderm in both assays (Figure S5G). Compared with the parental iPSCs, the ectoderm sample exhibited increased expression of neuroectodermal markers (*SOX2* and *NRG1*) and decreased expression of pluripotency markers (*POU5F1* and *FGF2*; Figure S5H). Similarly, the mesoderm cells showed an upregulated expression of mesoderm-associated markers (*PDGFRA, FOXF1*, and *WNT5A*). Gene signatures of each cell culture analyzed in STAMP-C were highly correlated with the scRNA-seq dataset generated on the same suspension of fixed iPSCs (Figure S5I).

### Sensitive detection of rare cell types

**Figure 6.**
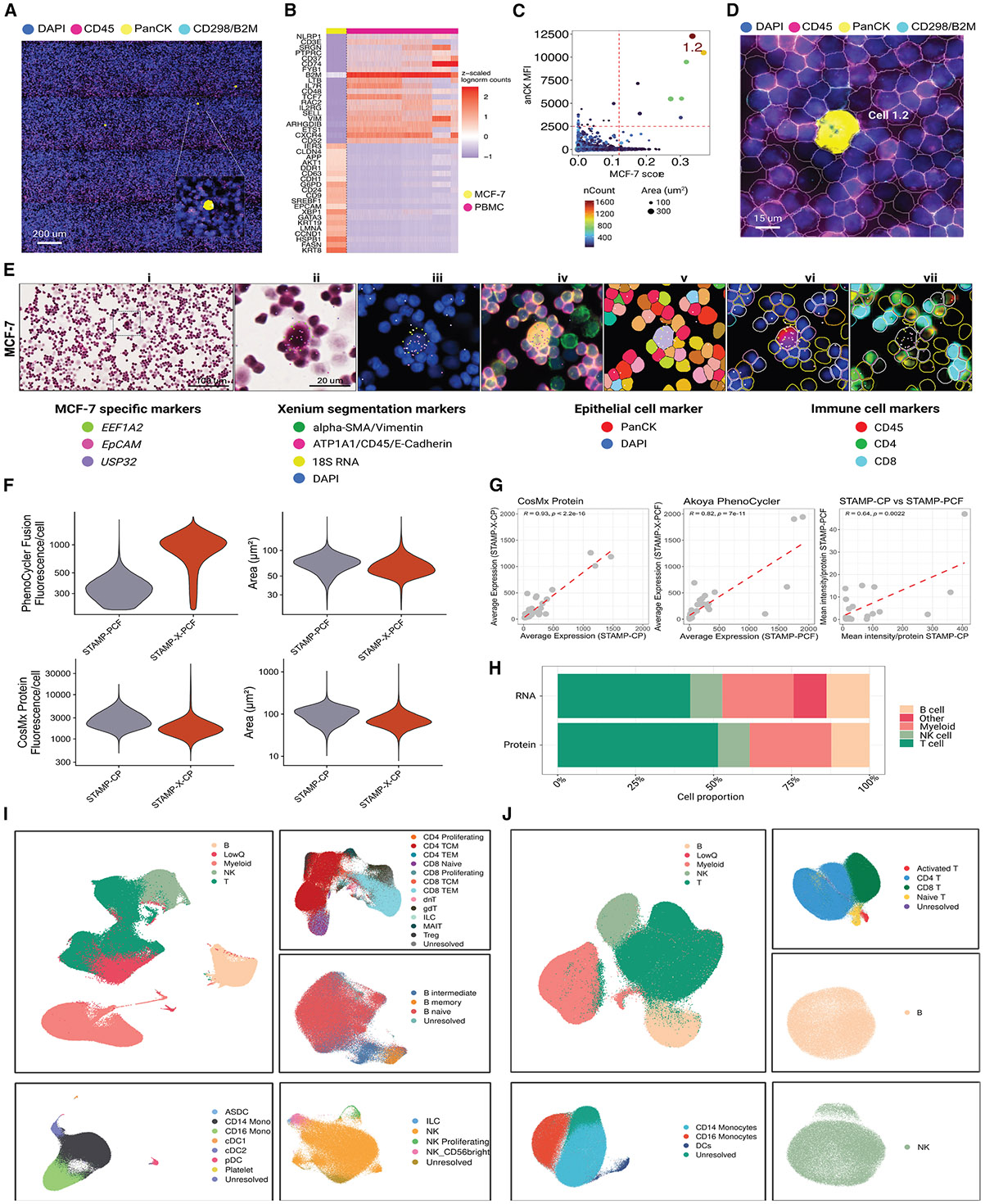
Circulating tumor cells mimics and multimodal analysis (A) Immunofluorescence image of a FOV with three CTCs (staining DAPI, PanCK, CD298/B2M, and CD45). (B) Heatmap of MCF-7 and PBMC gene signatures derived from differential expression analysis of the scRNA-seq dataset (subsetted to CosMx 1K panel). (C) Scatterplot of the MCF-7 signature score (as in B) and PanCK mean fluorescence intensity (MFI) of the 10 CTC-mimic. Each dot represents a cell (size proportional to cell area and color coded by the number of counts). (D) Immunofluorescence image highlighting a DAPI+ PanCK+ CD45− CTC-mimic (as in C; #1.2). (E) Multimodal visualization of a CTC-mimic identified in STAMP-X, including cell type markers and images of (i) H&E at low and (ii) high magnification, (iii) DAPI, (iv) Xenium cell segmentation, (v) segmented cells colored by cluster ID (Xenium Explorer), (vi) cell boundaries colored by cluster ID with PanCK staining, (vii) cell boundaries colored by cluster ID with immune cell marker staining. (F) Protein counts of PBMCs from CosMX and Phenocycler Fusion. Aggregated fluorescence per cell (left) and cell area (μm^2^, right). (G) Average expression of protein panels showing high correlation within and across platforms (MFI, mean fluorescence intensity; R and *p* value statistics from Pearson correlation). (H) Cell proportions detected by multimodal profiling of RNA and protein (STAMP-X/X-CP/CP and STAMP-X/X-PCF/PCF). (I) UMAP visualization of RNA STAMP-X-CP with unsupervised clustering and cell type annotations. (J) UMAP visualization of protein STAMP-X-CP with unsupervised clustering and cell type annotations. Related to Figure S6.

Given the scalable design of STAMP, we next aimed to simulate clinically relevant scenarios, particularly the identification of circulating tumor cells (CTCs). Tumor cells circulating in the blood are valuable for quantifying tumor burden, identifying tumor heterogeneity, and detecting actionable alterations. To simulate CTC detection from blood samples and approximate the sensitivity of STAMP in this experimental setting, we spiked-in MCF-7 cancer cells at dilutions of 1:100,000 and 1:50,000 into PBMC samples, stamped onto the same slide (total 1.1 million cells). We then imaged the cell mixtures with the CosMx Human Universal Cell Characterization RNA panel. Here, the CosMx cell segmentation markers provided the ground-truth, allowing for the visual detection of the CTC-mimics, which were randomly distributed across each sub-STAMP (PCK staining, Figure 6A). To automate CTC-mimic detection, we generated gene expression signatures using scRNA-seq performed on fixed cells and scored each imaged single cell based on their MCF-7 or PBMC profiles (Figure 6B). We additionally combined information of the PCK mean fluorescence intensity, the number of transcript counts, and the cell areas to jointly identify CTC-mimic with high accuracy (Figure 6C). Thereby, in sub-STAMPs with 10 and 20 target spike-in cells, we confidently identified 7 and 28 CTC-mimics, respectively. Here, CTC-mimics accounted for 0.001% of the overall sample size, demonstrating the capacity to detect extremely rare cellular types with clinical application potential. Of note, the immunofluorescence images allowed us to manually validate the identified CTC-mimics based on their coordinates registered with the STAMP experiments (Figure 6D). The detection of these rare events was independent of the platform, panel, or segmentation method, as demonstrated by the identification of CTC-mimics using the Xenium with the Immuno-Oncology panel (Figure S6A) and the Xenium Prime 5K Human Pan Tissue and Pathways panel (Figures 6E and S6B). Here, using the Xenium Explorer to align RNA, protein, and H&E images for visual integration, we identified and confirmed the presence of two cancer cells among 852,677 PBMCs in the sample (Figure S6B), demonstrating the sensitivity of STAMP in detecting rare cells.

### Combined RNA and protein multimodal profiling

Building on our scalable imaging-based approach for single-cell transcriptomics, we next integrated combined RNA and protein readouts into the STAMP framework for multimodal cell profiling. Therefore, we prepared two high-density STAMPs (~750,000 to 900,000 PBMCs) to generate and integrate RNA and protein imaging data. We first performed STAMP-X, using the Xenium Prime 5K Human Pan Tissue and Pathways panel for high-resolution RNA profiling, and then reused the same slides for subsequent protein profiling on two separate platforms: the CosMx SMI (64-protein panel; STAMP-X-CP) and the Akoya Phenocycler Fusion (40-protein panel, STAMP-X-PCF). Parallel single-modal protein STAMP experiments were conducted on the CosMx SMI (STAMP-CP) and the Phenocycler Fusion (STAMP-PCF) to assess the impact of prior STAMP-X transcriptomic processing on the protein profiling outputs.

All protein assays yielded high-quality protein profiles with substantial mean fluorescence intensity per cell, demonstrating the robustness of protein detection within the STAMP framework (Figure 6F). For STAMP-CP, we detected 582,087 cells with a mean fluorescence intensity of 1,430.7 and a mean cell area of 77.25 μm^2^, whereas in the STAMP-X-CP sample we detected 548,800 cells with a mean fluorescence intensity of 2,022 and a mean cell area of 73.62 μm^2^. For the Phenocycler, the STAMP-PCF sample contained 615,566 cells with a mean intensity of 350.8 and a mean cell area of 70.57 μm^2^, whereas the STAMP-X-PCF sample included 630,691 cells with a higher mean intensity (923.1) and a mean cell area of 65.17 μm^2^. To further evaluate the quality of multimodal profiling, we compared the average protein expression for each protein across STAMP-CP/X-CP and STAMP-PCF/X-PCF samples, i.e., with and without prior RNA profiling. Here, protein detection in both platforms correlated significantly (STAMP-CP/X-CP: R = 0.93, *p* < 0.01; STAMP-PCF/X-PCF: R = 0.82, *p* < 0.01; Figure 6G). It is important to note that significant differences were observed for specific proteins between the single- and multimodal workflows.

To evaluate the concordance between the two proteomics technologies, we quantified the mean fluorescence intensity of the shared proteins (*n* = 20). Pearson correlation analysis revealed a significant positive correlation (R = 0.64, *p* = 0.0022), where STAMP-CP exhibited higher overall mean fluorescence intensity (max = 407.35) compared with STAMP-PCF (max = 46.96). We next sought to compare the STAMP protein results from PBMCs of the CosMx (-CP, X-CP) and the PhenoCycler Fusion (-PCF, -X-PCF) to a sequencing-based readout (i.e., CITE-seq). The CITE-seq dataset included 228 antibodies, with 11 overlapping the CosMx and 20 overlapping the PhenoCycler panels. As expected, CITE-seq detected more features per cell due to its larger panel size, an effect that was mitigated when normalizing for the panel size or when analyzing specific lineage-defining marker proteins (Figures S7A and S7B). To validate that the RNA and protein data from single- and multimodal workflows could be integrated for immuno-phenotyping across modalities, we conducted cluster analysis of the PBMCs from multimodal STAMP-X-CP (Figures 6H-6J) and STAMP X-PCF (Figure S7C), identifying the major cell types across both modalities (Figures 6H-6J and S7C). Sub-clustering the transcriptome data of the major immune cell types revealed 26 subpopulations of T, B, and natural killer (NK) and myeloid cells (Figure 6I). Protein profiling identified the same major cell types but faced difficulties in describing more subtle cell states due to the smaller protein panels (Figure 6J).

### Profiling dissociated tissues with STAMP

**Figure 7.**
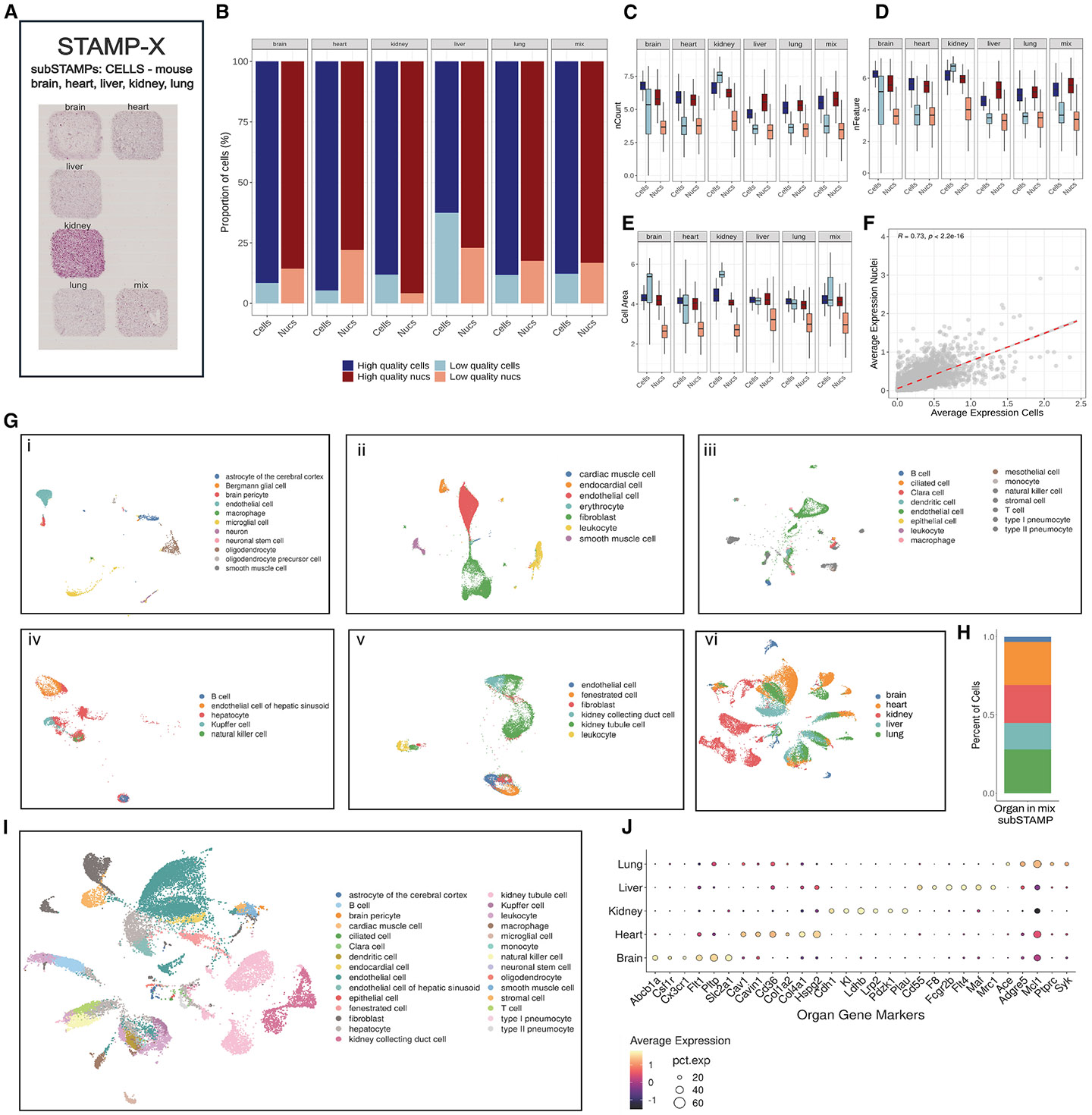
Profiling dissociated tissues with STAMP (A) Diagram of tissue STAMP design performed on cells dissociated from mouse tissues: brain, heart, kidney, liver, and lung—as well as a 1:1:1:1:1 mixture of cells from these organs. (B) Bar plot showing the proportion of high- and low-quality cells and nuclei for each mouse organ. (C) Boxplot showing the number (in natural log) of transcripts captured by tissue STAMP and cell isolation. (D) Boxplot of number of genes (in natural log) detected in cells from tissue STAMP across different organs. (E) Boxplot of the distribution of cell areas captured in tissue STAMP. (F) Scatterplot of average gene expression comparison between cells and nuclei across all tissue STAMP datasets. *p* < 0.05. (G) UMAP visualizations of cell clusters from individual mouse tissues: (i) brain, (ii) heart, (iii) lung, (iv) liver, (v) kidney, and (vi) a mixed cell population from all five organs. (H) Proportion of cells detected across organ types in the cell mixture. (I) UMAP visualization of all cell types identified in the mixed population from the five organs. (J) Dot plot highlighting the most highly expressed marker genes for each organ type. See also Figures S7E-S7H.

Despite the transformative potential of spatial transcriptomics, single-cell analysis of dissociated cells remains the gold-standard for characterizing tissues. To assess the versatility and applicability of STAMP for dissociated tissues, we performed tissue analyses of five major mouse organs: kidney, liver, brain, heart, and lung (Figures 7A and S7E-S7H). These organs were chosen for their diverse cellular compositions to provide an evaluation across various tissue architectures. For each organ, we prepared and processed both cell and nuclei suspensions. We included nuclei, as their preparation provides a less biased cell type coverage and avoids transcriptional artifacts associated with cell isolation, and for their compatibility with archived frozen specimens.^41^ Cells and nuclei suspensions were immobilized onto Xenium-compatible glass slides for STAMP-X analysis and profiled using the Xenium Prime 5K Mouse Pan Tissue & Pathways panel. Additionally, a portion of the fixed cells and nuclei was profiled using the Single-Cell Gene Expression Flex assay to compare quality and performance between STAMP and established scRNA-seq protocols. In total, 601,417 cells were profiled in two STAMP-X experiments (215,578 cells and 385,839 nuclei).

The STAMP data from cells and nuclei showed comparable frequencies of high-quality cells/nuclei and the numbers of detected transcripts and genes (Figures 7B-7E). We also observed a strong correlation between cells and nuclei across all organs (R = 0.73, *p* < 0.01; Figure 7F), with the liver being a notable exception for all metrics. In line with prior reports,^42-44^ isolating single cells from liver tissue presents significant challenges, primarily due to the organ’s complex architecture, the delicate nature of hepatocytes, and the limitations of current dissociation methods performed at a temperature at which liver enzymes are highly active. We also included brain single-cell and single-nuclei preparations due to the distinct biases to capture highly connected cell types, such as neurons. Here, applying STAMP on brain nuclei outperformed single-cell preparations, yielding a total of 6,862 cells and 45,487 nuclei, consistent with previous findings.^45,46^

Using the mouse reference cell atlas for high-level annotation,^47^ we identified 44 different cell types across all organs (Figure 7G). The lung dataset contained the highest number of cell types (*n* = 15), including various types of pneumocytes (Figure 7G, iii). In a sub-STAMP with a 1:1:1:1:1 mixture of cells from all organs, we computationally separated cell identities by applying gene signatures derived from each respective tissue (Figure 7I). In detail, we conducted a one-vs-all differential expression analysis for each organ and validated the organ-specificity of the top differentially expressed genes (Figure 7J).

## DISCUSSION

We developed STAMP, a cost-effective approach that transforms commercial imaging platforms into scalable and flexible profiling tools for transcriptomics and proteomics readouts of cells in suspension. STAMP provides a unified solution that integrates high-throughput analysis and multi-sample profiling, expanding the possibilities of single-cell analysis for diverse research and clinical applications. We evaluated STAMP with the main objectives of verifying (1) specificity, (2) reproducibility, (3) scalability, (4) sensitivity, (5) resolution, and (6) flexibility. Cancer cell lines were used to confirm specificity and to ensure the accurate targeting of gene sets within individual cells, without cross-hybridization or background noise. For reproducibility and scalability, we tested the analysis of millions of PBMCs across replicates, platforms, and gene panel sizes, demonstrating STAMP’s consistent performance. Flexibility was assessed through the profiling of cells and nuclei from fresh and preserved samples, together with various experiments multiplexing diverse cell and sample types. STAMP demonstrated adaptability to diverse sample sizes, cell types, and experimental conditions. Its sensitivity was proven in CTC-mimic experiments, detecting rare populations down to 0.00025%.

A critical advantage of the STAMP technology is its cost-effectiveness, providing a significant cost reduction compared with single-cell sequencing approaches. For example, a cost-efficient version of single-cell sequencing (10× Genomics; NextGEM RNA Flex, multiplexed with 4 barcodes) costs approximately USD 2,800 (including sequencing) for profiling ~10,000 cells per sample. A STAMP with 130 sub-STAMPs per slide (1 mm^2^ each) enables the profiling of ~20,000 PBMCs in each sub-STAMP for just USD 58. The cost advantage is even more pronounced in large-cohort studies. Analyzing 1,000 PBMC samples at 25,000 cells/sample costs USD 3.56 million using single-cell sequencing, whereas STAMP achieves the same scale at USD 75,000. This 47-fold reduction relates to USD 0.14/cell for single-cell sequencing and USD 0.003/cell with STAMP. Furthermore, STAMP offers significant cost efficiency in feature detection. Although single-cell sequencing detects approximately 1,200–1,800 features/cell for immune cells, STAMP identifies 600–800 features/cell (Xenium Prime 5K targeted imaging panel), reducing the costs per feature from USD 1.98–2.96 to USD 0.093–0.125.

Despite being more cost-efficient, STAMP has comparable sensitivity in gene detection compared with single-cell assays when analyzing intersecting gene sets. Benchmarking of the non-specialized Xenium panel against 3′, 5′, and Flex sequencing assays showed similar capability to detect transcripts and genes across the main immune cell lineages. However, sequencing assays were still more sensitive considering a transcriptome-wide analysis. Refining or expanding probe sets would further improve resolution for an even more precise mapping of cellular heterogeneity and cellular trajectories as the basis for large-scale gene and drug perturbation studies. The multimodal integration highlights STAMP’s potential as a powerful tool for multi-layered data analysis, where RNA and protein information can be combined to improve phenotype and functional mapping. By replacing sequencing with imaging, STAMP also captures spatially indexed morphology data, supporting visual inspection to reduce false positive results and enabling multimodal integration of RNA, protein, and morphometrics. STAMP could also facilitate the visual validation of receptor-ligand interactions, an application that, however, requires optimization of the currently applied dissociation and sample preparation protocols.

### Limitations of the study

Although STAMP provides cost-effective and scalable single-cell transcriptomics, it has inherent limitations common to imaging-based methods. Its reliance on pre-defined gene panels restricts novel transcript discovery, though customization options will help expand gene coverage. Probe-based detection can introduce hybridization biases, potentially skewing gene expression profiles, which can be mitigated through optimized probe design and computational normalization. STAMP is unsuitable for full-length transcript analysis, limiting its ability to detect alternative splicing events. SNP, mutation, and immune receptor profiling (TCR/BCR) are also challenging due to probe design constraints and high sequence variability. Additionally, while boutique CRISPR Perturb-seq applications are possible, pooled CRISPR screens are currently not well supported, though future developments may address this limitation. Despite these challenges, STAMP integrates scalability, cost-effectiveness, and flexibility into a single approach. This enables researchers to perform experiments on a broad range of sample sizes, from limited biopsy specimens to extensive cellular atlasing, without compromising data quality or increasing costs.

## STAR★METHODS

### EXPERIMENTAL MODEL AND STUDY PARTICIPANT DETAILS

#### Maintenance of human cell lines

All cell lines used in this study were cultured and maintained under the conditions outlined in Table S1. Cell counting and viability measurements, both before and after storage, were performed using the Luna-FX7 Automated Cell Counter (Logos Biosystems) with the AO/P Viability Kit (F23011). Cell viability after thawing was consistently >85%. On the day of fixation, cells were thawed in a 37 °C water bath and washed twice with 1x PBS (without Ca^2+^/Mg^2+^) supplemented with 0.05% BSA (Miltenyi Biotec, 130-091-376). For the tongue cancer-associated fibroblasts, sample collection was conducted with informed patient consent and approved by the Central Adelaide Local Health Network Human Research Ethics Committee (approval number: 17981).

### TISSUE COLLECTION AND PREPARATION

Heart, brain, liver, kidney, and lung tissues from scavenged C57Bl/6 female mice (5 month) were obtained from Dr. Hon Yeung Chan, Robertson Lab, The University of Adelaide. The mice were humanely killed by cervical dislocation under the approval of the Adelaide Animal Ethics Committee (approval number: M-2019-087). The scavenged organs were harvested within 10 minutes and immediately immersed in cold 1x PBS to maintain cellular integrity. Cells and nuclei were prepared as follows. To create single-cell suspensions, tissues were mechanically dissociated into smaller fragments and subjected to enzymatic digestion using the gentleMACS Octo Dissociator with Heaters (Miltenyi Biotec, 130-096-427). The enzyme used for digestion was Liberase TH (Roche, 792347) at a concentration of 0.5 mg/mL for 30 min at 37 °C (program 37C_Multi_G). Following digestion, cell suspensions were filtered through a 40 μm strainer to obtain a pure single-cell suspension. Cell viability was assessed using the Luna FX7 to ensure suitability for single-cell analysis. Nuclei suspensions were prepared following the *SaltyEz protocol* as described at https://doi.org/10.17504/protocols.io.bx64prgw. For the FFPE prostate samples (31944 1N and 31568 F), ethical approval for tissue collection and experimentation was obtained from the Royal Adelaide Hospital (approval #041011f) and the University of Adelaide Human Research Ethics Committees (approval number: H-2018-222).Differentiation of Human Embryonic Stem Cell (hESC)Human embryonic stem cells (hESCs, WA01, WiCell) were cultured in vessels coated with growth factor-reduced Matrigel (Corning, 354230) and maintained in mTeSR+ growth media (STEMCELL Technologies, 85850). Cultures were passaged by removing the medium, washing with DPBS (Sigma-Aldrich, D8537), and treating with Gentle Cell Dissociation Reagent (STEMCELL Technologies, 100-0485,) at sufficient volume to cover cells for 3 min at 37 °C. The dissociation reagent was aspirated, growth media was added, and cells were lifted by scraping with a cell lifter to produce small cell clumps. One-sixth volume of lifted cells was added to a new culture vessel and topped up with mTeSR+ growth media. All cultures were grown at 37 °C, 5% CO_2_. For differentiation, hESCs were seeded as colonies into 6-well trays by passaging as described. The following day, hESCs were treated with 50 ng/mL human recombinant BMP4 (STEMCELL Technologies, 78211) in mTeSR+ media for 0, 6, 12, 24, 48, 72, 96, or 120 h, with daily media replacement. To harvest, media was removed, cells were washed with DPBS, and Gentle Cell Dissociation Reagent was added to cover the cells. Cultures were incubated at 37 °C until dissociated, then growth media was added. Dissociated cells were pelleted by centrifugation for 5 min at 200 rcf, and the media was aspirated. Cell pellets were resuspended in 1 mL mFreSR (STEMCELL Technologies, 05855) and transferred to a cryotube, then frozen in a CoolCell Container (Corning) at −80 °C overnight. Cells were transferred to −150 °C storage the next day. Cell counting and viability measurements before and after storage were carried out using the Luna-FX7 Automated Cell Counter. Viability of cells after thawing was >85%.

#### Differentiation of induced pluripotent stem cells and Immunofluorescence

Induced pluripotent stem cells (iPSCs, 32F) were generated via Sendai virus reprogramming as previously described^49^ and maintained in E8 media (ThermoFisher Scientific, A2656101) on Matrigel-coated plates (Corning, 354277). For mesodermal differentiation, iPSCs were seeded at 90% confluency in E8 medium. On day 1, cells were switched to RPMI (ThermoFisher Scientific, 12633012) supplemented with 0.5% B27 without insulin (ThermoFisher Scientific, A1895601) and 40 ng/mL BMP4 (PeproTech, 120-05ET-500UG). On day 2, the medium was replaced with the same medium plus 6 μM CHIR99021 (STEMCELL Technologies, 72052). On day 3, 5 μM IWR1 (STEMCELL Technologies, 72562) was added, removed the next day, and replaced with RPMI + 0.5% B27 without insulin. For ectodermal differentiation, iPSCs were plated in E8 medium until 80% confluency. On day 1, the medium was replaced with neural induction medium (NIM) composed of DMEM/F12 (ThermoFisher Scietific, 11320033) + 1x NEAA (ThermoFisher Scientific, MEM Non-Essential Amino Acids, 11140050) + 0.5% B27 (ThermoFisher Scientific, 17504044), 5 uM SB431542 (STEMCELL Technologies, 100-1051), and 0.1 uM LDN193189 (Sigma Aldrich, SML0559) for 5 days, after which cells were harvested. For endodermal differentiation, cells at 50% confluency were switched to RPMI supplemented with 0.5% B27 without insulin, 100 ng/mL Activin A (PeproTech, 120-14E), 10 ng/mL BMP4, and 20 ng/mL FGF2 (PeproTech, 100-18B). On day 3, cells were switched to RPMI supplemented with 0.5% B27 without insulin and 100 ng/mL Activin A until harvest on day 5. Cells were harvested using Accutase (ThermoFisher Scientific, A1110501), centrifuged for 2 min at 400 rcf, and washed twice with PBS (without Ca^2+^/Mg^2+^) + 0.05% BSA. Viability at harvesting was >90% as measured using the Luna-FX7 Automated Cell Counter. All cultures were grown at 37 °C, 5% CO_2_. Cells were processed for STAMP on the same day of collection. Expression of canonical marker genes for all embryonic lineages was assessed as part of sample quality control. Based on the absence of marker gene expression (SOX17, HNF4A, GATA6, PRDM1) it was concluded that iPSCs in the Endoderm sample had failed to sufficiently differentiate towards endoderm. This sample was therefore excluded from further analysis. For immunofluorescence analysis, cells of all lineages were seeded on Matrigel-coated chamber slides (ThermoFisher Scientific, Nunc-Labtek, 171080), fixed with 4% PFA for 15 min at room temperature (RT), and permeabilized with 0.2% Triton X-100 (ThermoFisher Scientific, A16046.AE) in PBS for 10 min. Cells were blocked with PBS (without Ca^2+^/Mg^2+^) + 1% BSA and 0.2% Triton X-100 for 30 min at RT, followed by incubation with the primary antibody in blocking buffer overnight at 4 °C. After washing three times with blocking buffer, cells were incubated with secondary antibodies at RT for 1 h in the dark, washed three times with blocking buffer, and stained with 1x DAPI (ThermoFisher Scientific, R37606). Confocal images were acquired using an Olympus FV3000 Confocal Microscope. Analysis was performed using ImageJ software (U.S. National Institutes of Health, Bethesda, Maryland, USA). The primary antibodies used were: GATA4 (G-4, sc-25310), Nestin (SAB4200347), Pax6 (AMAB91372), SOX2 (14-9811-82), Oct3/4 (C-10, sc-5279), and TRA-1-60 (MA1-023).

#### Activation of PBMCs

Cryopreserved −80°C Human Peripheral Blood Leukopak (PN 200-0132, Stem Cell) were thawed in a 37°C water bath and transferred with a bored tip to a 15 mL Falcon containing 14 mL of 37°C pre-warmed RPMI (L-Glutamine) media (PN 11875093, Thermo Fisher Scientific) supplemented with 10% FBS (PN 16140071, Thermo Fisher Scientific) and 100 U/mL Penicillin/Streptomycin (PN 15140122, Gibco). PBMCs were centrifuged at 300x g for 7 min at RT, supernatant was removed, and pellet resuspended in 1 mL of 1X PBS (Thermo Fisher Scientific) supplemented with 1% BSA (PN 130-091-376, Miltenyi Biotec) and 10 μL of DNase I (PN LS002007, Worthington-Biochem). After incubation at RT for 10 min with periodical shaking, cells were filtered with a 40 μm strainer (PN 43-10040-40, Cell Strainer) into a new 15 mL falcon on ice and filter was washed by adding 9 mL of cold 1X PBS. PBMCs were spinned down by centrifuging at 300x g for 7 min at 4°C and resuspended in 1X PBS with 0,05% BSA for assessment of cell numbers and viability with the TC20^™^ Automated Cell Counter (PN 1450102, Bio Rad), obtaining a viability >85%. PBMCs were then cultured in a 6 well plate at 4 million cells/mL in complete RPMI media at 37°C + 5% CO2 under three different culture conditions: (1) Dynabeads^™^ Human T-Activator CD3/CD28 for T Cell Expansion and Activation (PN 11132D, Thermo Fisher Scientific) following manufacturer’s protocol, (2) lipopolysaccharide (LPS) at 100 ng/mL (PN L2630-25MG, InvivoGen); and (3) complete medium only as control. After 4 and 24 h in culture, cells in each condition were harvested, washed with filtered 1X PBS with 0,05% BSA and STAMPed as previously described.

### METHOD DETAILS

#### Slide Preparation and “STAMPing” Procedure

Superfrost Plus Micro Slides (VWR, 48311-703) for STAMP-C/CP/PCF and Xenium slides (10x Genomics, PN-3000941) for STAMP-X/X-CP/X-PCF/PCF, were placed on Xenium slide holders were coated with 1 mL of stock Poly-D-Lysine for 1 h and overnight at 37 °C, respectively, in a thermocycler using the Xenium Thermocycler Adapter plate positioned atop the 96-well block of a C1000 Touch Thermal Cycler (BioRad) with the lid closed and set at 37 °C. After coating, the slides were washed with 1 mL of Nuclease Free Water (ThermoFisher Scientific, 10977023) 3 times and air dried. Custom single or multi-plex (multi-sample) arrays of various areas/volumes were created using either 10x Genomics’ gaskets (PN- 370017) from the Chromium Single Cell Reagent Kits and using a hole punch or punch pliers (Total Tools, 9070220SB), micro-Slide 8-well (ibidi, 80841) and 12-well (ibidi, 81201) cell culture chambers or a silicone gasket for ProPlate^®^ microarray system (Grace Bio-Labs, 246875) by placing them on the coated slides within the scanning area of the CosMx, Xenium or Phenocycler. For the STAMPing procedure (i.e. attaching cells on slides for STAMP), up to 5 million single cells/nuclei in suspension, with >80% viability, were first fixed with 4% Formaldehyde (Sigma-Aldrich, 252549-500ML) + 1x Concentrated Cell Fixation and Permeabilization Buffer (10x Genomics, PN-2000517), as per the Fixation of Cells & Nuclei for Chromium Fixed RNA Profiling protocol (CG000478, RevD). After 2 h incubation at RT, cells were pelleted at RT for 5 min at 850 rcf and resuspended in 1 mL of 1x Quenching Buffer (10x Genomics, PN-2000516), washed 2 times with nuclease-free water and finally resuspended in water + 0.01% Triton-X and counted in duplicates or triplicates using the Luna-FX7 Automated Cell Counter. To maximize the use of the area in each stamp (or substamp, when in multi-sample settings) for high and ultra-high cell profiling we used the average cell size data provided by the Luna-FX7 Automated Cell Counter and estimates generated using ChatGPT 4o to calculate the number of cells that would fully (or partially) cover a desired area of the given custom array. Before STAMPing, cell concentrations were adjusted so that the minimum volume of cell suspension added to the wells would evenly cover the bottom surface for the desired number of cells to profile and fully dry within 1 h of incubation at 42 °C. This volume was dependent on the well area and was approximated in advance for each well size. Cells were then loaded into the wells, and the slide was placed in a thermocycler using the Xenium Thermocycler Adapter plate, running the following program: 4 °C for 30 min, 25 °C for 5 min, 42 °C for up to 1 h (or until the volume dried), followed by 42 °C for 2 h, and then held at 22 °C. After STAMPing, custom wells were carefully removed, and the slides were either processed immediately or placed in a mailer with desiccant at RT for STAMP-X/X-CP/X-PCF or at 4 °C for STAMP-C/CP/PCF until further processing. The same procedure was carried out for STAMP profiling using PBMCs from a healthy donor (STEMCELL Technologies, 200-0470), human cell lines (MCF-7, SK-BR-3, LNCaP, EndoC-betaH1, hTERT-HME1, TF-1, U-373 MG, UMSCC-1, V16D, CHP-134, HEK293T, SK-N-DZ, SK-N-SH, SHSY-5Y and KELLY either individually or mix 1:1:1 MCF-7:SK-BR-3:LNCaP or MCF-7:SK-BR-3:LnCAP:KELLY), iPSCs, iTSCs, Human dermal fibroblasts, hESC, Tongue Cancer CAFs, BM CD34+ (STEMCELL Technologies, 70002), nuclei isolated from FFPE prostate tumour samples 31944 1N and 31568 F, cells and nuclei isolated from mouse heart, lung, brain, liver and kidney and CTC-mimics. For CTC-mimics, MCF-7 and SK-BR-3 cell lines were counted in triplicates and spiked into 1 million PBMCs either as a single line (MCF-7) or as a mix (1:1 MCF-7:SK-BR-3). Spike-in ratios varied from less than 10 to more than 10 but fewer than 50 cancer cells per approximately 1 million PBMCs.

#### CosMx RNA Slide Preparation (STAMP-C)

STAMPed slides were placed on a Xenium Thermocycler Adapter plate atop the 96-well block of a thermocycler with the lid open and incubated at 60 °C for 2 h, then equilibrated to RT for 3–5 min. The slides were subsequently processed according to the guidelines provided in the CosMx SMI Slide Preparation for FFPE RNA Assays manual (NanoString, MAN-10184-02 or MAN-10184-03 for 6k), starting from page 38 or 36 respectively. Slides were immersed directly in pre-heated 1x Target Retrieval Solution (NanoString, a Bruker company CosMx FFPE Slide Preparation RNA Kit) in a pressure cooker at 100 °C for 8 min (as per MAN-10184-02 and MAN-10184-03). The slides were immediately transferred to water for 15 s, then washed in 100% ethanol for 3 min, and air-dried at RT for 30 min to 1 h. Incubation frames were attached to each slide, and a pre-warmed digestion buffer containing 1.5 μg/mL Proteinase K (NanoString, CosMx FFPE Slide Preparation RNA Kit) and 1x PBS (ThermoFisher Scientific, AM9625) was applied. Slides were incubated in a hybridization chamber at 40 °C for 15 min. The slides were then rinsed twice in water, and fiducials were applied at 0.001%, followed by a 5 min incubation at RT, shielded from light. The slides underwent a 1x PBS wash for 1 min, followed by fixation in 10% NBF for 1 min, and then two washes in NBF stop buffer (Tris Base, Sigma S6639-1L) and Glycine (Sigma, G7126) for 5 min each, and a 5 min wash in 1x PBS. A 100 mM NHS-acetate solution (ThermoFisher Scientific, 26777) was applied to the tissue for 15 min at RT, followed by two washes in 2X SSC (ThermoFisher Scientific, AM9763) for 5 min each. The CosMx Human Universal Cell Characterization RNA Panel targeting 950 human genes, and a 50-target add-on panel set (NanoString, CMX-H-USCP-1KP-R), or the CosMx 6k Discovery Panel (Nanostring, 121500041), were denatured at 95 °C for 2 min, cooled on ice for 1 min, and then added to a probe mix containing RNase inhibitor, Buffer R, and nuclease-free water. This mix was applied to the slide and incubated for 18 h in the hybridization chamber at 37 °C. After incubation, the slides were washed twice in a final concentration of 50% deionized formamide (ThermoFisher Scientific, AM9342) and 2x SSC mix for 25 min each, followed by two washes in 2x SSC for 2 min each. DAPI nuclear stain stock was diluted to 1:40 with a blocking buffer (Nanostring, CosMx FFPE Slide Preparation RNA Kit) and applied to the slides for 15 min at RT, protected from light. The slides were then washed in 1x PBS for 5 min and stained for 1 h with a cocktail of CD298, B2M, PanCK, and CD45 antibodies (Nanostring, 121500020, 121500021). The slides were washed three times in 1x PBS for 5 min each and then stored in 2X SSC. The pre-bleaching profile followed configuration A, while the cell segmentation profile adhered to configuration C (MAN-10161-03-2 or MAN-10161-05, Nanostring).

#### CosMx Protein Slide Preparation (STAMP-X-CP/CP)

STAMPed slides (for both single and multimodal STAMP) were processed according to the guidelines provided in the CosMx SMI Slide Preparation for FFPE Protein manual (MAN-10185-01-1, NanoString) from page 31. Briefly, slides were baked at 65 °C for 2 h, equilibrated to RT for 3–5 min, then rehydrated in 1x PBS for 5 min. Slides were immersed in pre-heated 1x Target Retrieval Solution (Nanostring, CosMx Protein Slide Preparation FFPE Kit) in a pressure cooker at 100 °C for 8 min and then allowed to equilibrate to RT in the same solution for 60 min. Subsequently, the slides were washed three times in 1x PBS for 5 min each, and incubation frames were attached. Slides were then covered with Buffer W and incubated at RT for 1 h in the dark. The 64-plex Human Immuno-Oncology Protein (antibody) Panel (Nanostring, CMX-H-IOP-64P-P) was combined with CD298, B2M, PanCK and CD45 segmentation markers in Buffer W. The primary antibody mix was incubated at 4 °C for 18 h, followed by three washes with 1x TBS-T buffer for 10 min each and a wash with 1x PBS for 2 min. Fiducials, prepared at the recommended concentration of 0.00005%, were applied to the slides at RT for 5 min, protected from light. Slides were then washed once with 1x PBS for 5 min and fixed with 4% PFA for 15 min, followed by three washes in 1x PBS for 5 min each. Sections were stained with a 1:40 diluted nuclei stain for 10 min, washed twice with 1x PBS for 5 min, and incubated with 100 mM NHS acetate for 15 min, before a final wash in 1x PBS for 5 min. The selected pre-bleaching profile was Configuration A, and the cell segmentation profile was Configuration C.

#### CosMx SMI Setup and Data Acquisition (STAMP-X-CP/CP/C)

The setup and scan acquisition for the CosMx SMI instrument were performed according to the CosMx SMI Instrument User Manual (MAN-10161-03-2 or MAN-10161-05 for 6k, Software Version 1.3.0.209, NanoString). A new acquisition process was initiated through the CosMx SMI Control Center web interface. Before insertion into the CosMx Flow Cell Assembly Tool, the slides were carefully dried in the areas surrounding the imaging region. The assembly process involved lowering the tailgate, placing the slide, and securing it by raising the tailgate. After removing the adhesive backing, a new flow cell coverslip was precisely aligned above the slide’s imaging area. The Assembly Tool’s lid was then closed securely to attach the coverslip to the slide, creating a functional CosMx flow cell. Following assembly, 2x SSC (for RNA assay) or 1x PBS (for protein assay) was gently introduced through one of the flow cell ports to hydrate the samples. For multi-sample STAMPs, prior to placement of the flow cell, each STAMP was hydrated with 4 ul of 2x SSC (for RNA assay) or 1x PBS (for protein assay). The flow cell coverslip was slowly lowered from left to right allowing the liquid to spread evenly across the stamps. A P10 tip was used to help guide the coverslip down. Once positioned, the tip was removed and the assembly tool closed to secure the flow cell. This method prevented the formation of “rivers” inside the flow cell chamber and ensured full sample hydration.

Flow cell configuration data, including the flow cell’s barcode, slide ID number, and the maximum tissue thickness of 7 μm, were entered into the Control Center interface (MAN-10161-03-2 or MAN-10161-05, Nanostring). All slides were scanned using Configuration A for the pre-bleaching profile and Configuration C for the cell segmentation profile. Additional details regarding the probe panel, cell segmentation, and supplemental markers were also entered into the flow cell configuration data for each section. Assembled flow cells/slides, along with Buffer Bottles 1-4, were loaded into the instrument. Before positioning Bottle 4, catalase and pyranose oxidase enzymes were added directly before loading, or added the previous day for 6k runs. For RNA runs, RNase inhibitor was added to a designated well in the CosMx imaging tray, which was placed in the instrument after equilibration to RT. The Control Center configuration was verified, followed by a pre-run check and a tissue find scan for each slide conducted by the instrument. After completing the tissue find scans, rectangular scan areas were placed around each tissue section for the preview scan. The preview scan images enabled the selection of regions of fields of view (FOVs), ensuring thorough coverage of the STAMP areas on each slide. FOV selections for each slide were confirmed before starting the cycling process. CosMx scan data was automatically uploaded to NanoString’s cloud-based AtoMx Spatial Analysis Platform during the run, as detailed in the CosMx Data Analysis Manual (MAN-10162-03, Software Version 1.3.2, NanoString). Upon completion of the run and full upload of scan data to AtoMx, a study was created for each CosMx scan. Within AtoMx, pipelines were executed for each study, and data was exported in various formats, including TileDB arrays and Seurat objects, for in-house analysis.

#### Xenium Slide Preparation (STAMP-X)

STAMPed Xenium slides were placed on a Xenium Thermocycler Adapter plate atop the 96-well block of a thermocycler with the lid open and incubated at 60 °C for 2 h for Xenium v1 or 30 min (cells) or 1 h (dissociated tissues) for Xenium Prime. Slides were then equilibrated to RT for 7 min, assembled into Xenium cassettes, and hydrated with 1x PBS for Xenium v1 or PBS-T or 1X PBS for Xenium Prime. The slides were processed following the Xenium In Situ for FFPE Deparaffinization and Decrosslinking protocol from step 1.4.a (page 42) (CG000580 Rev D or Rev E, 10x Genomics). Briefly, the slides were reverse crosslinked using a decrosslinking buffer containing tissue enhancer, urea, and perm enzyme B at 80 °C for 30 min, followed by three washes with PBS-T. For Xenium v1, slides were then immediately processed according to the Xenium In Situ Gene Expression Cell Segmentation User Guide (10x Genomics, CG000749 Rev A) for the remaining slide preparation steps. Briefly, the pre-designed gene expression probe set, Xenium Human Immuno-oncology Panel (10x Genomics, PN-1000654), targeting 380 human genes, was denatured at 95 °C for 2 min, crash-cooled on ice for 1 min, and then equilibrated to RT before being added to the probe hybridization buffer and TE buffer (Fisher Scientific, BP24731) to make the probe hybridization mix. The slides were incubated with the hybridization mix at 50 °C for 20 h. Slides were washed three times with PBS-T for 1 min each and then incubated with a post-hybridization wash buffer for 30 min at 37 °C. Slides were washed three times with PBS-T for 1 min each and incubated with the ligation mix for 2 h at 37 °C. Three 1 min PBS-T washes were followed by a 2 h incubation at 30 °C with amplification mix and enzyme (10x Genomics, PN-2000392, 2000399). Slides were then washed three times with TE buffer for 1 min each, 70% ethanol for 2 min, twice with 100% ethanol, and once with 70% ethanol for 2 min each before rehydration with PBS-T. Slides were incubated at RT for 1 h with 1x Xenium Block and Stain Buffer (10x Genomics, PN-2001083). For segmentation staining, slides were incubated for 20 h at 4 °C with the Xenium Multi-Tissue Stain Mix (10x Genomics, PN-2000991), which contains a cocktail of antibodies labeling the membranes (anti-ATP1A1/CD45/E-cadherin), antibodies labeling the cell interior (anti-alphaSMA/Vimentin), and a universal interior label against Ribosomal RNA (18S rRNA) (10x Genomics, PN-2000991). Staining was enhanced by the addition of Xenium Staining Enhancer reagent (10x Genomics, PN-2000992), followed by treatment with Xenium Autofluorescence Mix (10x Genomics, PN-2000753) to diminish unwanted autofluorescence and enhance the signal-to-noise ratio. Subsequently, Xenium Nuclei Staining Buffer (10x Genomics, PN-2000762) was used to facilitate the identification of tissues or regions of interest during the instrument’s overview scan. For Xenium Prime, slides were immediately processed following the Xenium Prime In Situ Gene Expression with Optional Cell Segmentation User Guide (CG000760 Rev A, 10x Genomics) for the remaining slide preparation steps. Briefly, following decrosslinking steps in CG000580, Xenium 5K Human or Mouse PTP Panel Priming Oligos (10x Genomics, PN-2001224 or 2001226) were denatured at 95 °C for 2 min, crash-cooled on ice for 1 min, and then equilibrated to RT before being added to Priming Hybridization Mix with TE buffer and Priming Hybridization Buffer (10x Genomics, PN-001228). A Xenium Cassette Insert was placed onto the Xenium Cassette v2, and the slides were incubated with the priming hybridization mix at 50 °C for 1.5 h, then washed twice with PBS-T and incubated with Post-Priming Wash Buffer (10x Genomics, PN-2001229) at 50 °C for 30 min. Following three 1 min PBS-T washes, slides were incubated with an RNAse mix containing 2X RNAse buffer, RNase enzyme (10x Genomics, PN-2000411, 3000953), and water at 37 °C for 20 min. Slides were then washed three times with 0.5X SSC-T and incubated with a polishing reaction mix containing Polishing Buffer, Polishing Enzyme (10x Genomics, PN-2001231, 2001230), and water at 37 °C for 1 h. The pre-designed gene expression probe set, Xenium Prime 5K Human or Mouse Pan Tissue & Pathways Panels (10x Genomics, PN-1000724, 1000725), was denatured at 95 °C for 2 min, crash-cooled on ice for 1 min, and then equilibrated to RT before being added to the probe hybridization buffer and TE buffer to make the probe hybridization mix. Following three 1 min PBS-T washes, Xenium Cassette Inserts were placed onto the Xenium slide, and the slides were incubated with the hybridization mix at 50 °C for 20 h. Slides were washed twice with PBS-T for 1 min each, then incubated with a post-hybridization wash buffer (10x Genomics, PN-2000395) for 15 min at 35 °C. Slides were washed three times with PBS-T for 1 min each and incubated with ligation mix containing ligation enzymes A, B, and ligation buffer (10x Genomics, PN-2000397, 2000398, 2001233) for 30 min at 42 °C. Following ligation, slides were washed three times with PBS-T for 1 min each and incubated with Amplification Enhancement Master Mix containing Amplification Enhancer Buffer and Amplification Enhancer (10x Genomics, PN-2001234, 2001235) at 4 °C for 2 h. Amplification Enhancer Wash Buffer (10x Genomics, PN-2001236) was added, and slides were incubated with amplification mix (10x Genomics, PN-2000392) at 30 °C for 1.5 h, followed by three 1 min washes with TE buffer. Slides were processed for segmentation staining as outlined above for Xenium v1.

#### Xenium Analyzer Setup and Data Acquisition

STAMPed Xenium slides, assembled within Xenium cassettes, were imaged using the Xenium Analyzer in accordance with the guidelines specified in the Xenium Analyzer User Guide (CG000584 Rev F, 10x Genomics). The Xenium Decoding Consumables Kit (10x Genomics, PN-1000487) was used for instrument loading. Briefly, Xenium slide ID numbers and information on pre-designed gene expression probes were input into the Analyzer, and the necessary consumables and reagents were loaded into the instrument. Reagent modules B, and C for Xenium v1, were thawed at 4 °C overnight and equilibrated to RT for 30 min before loading into the instrument, while reagent module A was stored at 4 °C until loaded. Instrument wash buffer (100% Milli-Q water), sample wash buffer A (1x PBS, 0.05% Tween-20 (ThermoFisher Scientific, 28320), sample wash buffer B (100% Milli-Q water), and probe removal buffer (50% DMSO (Sigma Aldrich, D8418) 50 mM KCl (ThermoFisher Scientific, AM9640G), 0.1% Tween-20) were prepared and loaded into the instrument along with buffer caps, a pipette tip rack, an extraction tip, and the objective wetting consumable. After loading, the samples were scanned to generate images of the fluorescently labeled nuclei in each section, which were used for Field of View (FOV) selection prior to run initiation. Each STAMP area was selected as a separate region and labeled accordingly. Upon completion of the run, the instrument was cleared of consumables, and the Xenium slides were carefully removed. Fresh PBS-T was applied to each slide/cassette, which were then covered and stored in the dark at 4 °C for up to 3 days until post-run H&E staining. The data were acquired using the Xenium Explorer software suite (v3.1.0, 10x Genomics), which provides a set of applications for analyzing and visualizing in situ gene expression data produced by the Xenium Analyzer.

#### From Xenium RNA to CosMx Protein (STAMP-X-CP) and PhenoCycler-Fusion *(STAMP-X-PCF)*

Following the completion of the Xenium Prime 5k Human RNA run, slides were removed from the instrument and stored in the Xenium cassette with 50% glycerol in 1x PBS at 4 °C. After 6 days of storage, one of the slides (STAMP-X-CP) was washed three times in 1x PBS for 1 min each, followed by two additional washes in 1x PBS for 5 min each. The Xenium cassette was then removed, and the slide was placed into a wash jar containing 1x PBS for 5 min, as described in the CosMx SMI Manual Slide Preparation for Protein Assays (MAN-10185-01-1, NanoString). The slide was then immersed in antigen retrieval solution and incubated for 8 min at 100 °C, according to the CosMx Protein Slide Preparation and CosMx SMI Setup and Data Acquisition protocols. The other slide (STAMP-X-PCF) was covered with 50% glycerol in 1x PBS, coverslipped and sealed for shipment to The Jackson Laboratory (CT, USA) for PhenoClycler Fusion profiling (see below).

##### PhenoCycler-Fusion Slide Preparation (STAMP-X-PCF/PCF)

The STAMP-PCF slide was baked at 60 °C for 30 min. During the final 5 min of baking, the STAMP-X-PCF slide was removed from 50% glycerol/PBS storage and placed in 1x PBS. After baking, both slides were washed twice in 1x PBS for 5 min each. The slides were then immersed in 1x antigen retrieval buffer (pH 9.0) (AR9, Akoya Biosciences), and antigen retrieval was performed at 95 °C for 8 min using the TintoRetriever (BioSB). Following antigen retrieval, the slides were cooled in the retrieval buffer to RT and washed in nuclease-free water for 5 min. Slides were then processed according to the PhenoCycler-Fusion User Guide (PD-000011 REV M, Akoya Biosciences), starting from step 4 on page 49. Briefly, the slides were washed in Hydration Buffer, equilibrated in Staining Buffer, and incubated overnight at 4°C with a 40-marker antibody cocktail (Table S2) prepared in Blocking Buffer. The slides were subsequently washed in Staining Buffer, gently fixed with Post-Staining Fixing Solution, washed in 1x PBS, and incubated in ice-cold methanol for 5 min. The slides were then washed in 1x PBS, fixed with Final Fixative Solution for 20 min, washed three times in 1x PBS, and immersed in Storage Buffer prior to the PhenoCycler Fusion run.

##### PhenoCycler Setup and Data Acquisition (STAMP-X-PCF/PCF)

The experimental protocol was set up using the PhenoCycler Experiment Designer (Version 2.1.0, Akoya Biosciences). A reporter plate containing fluorescently labeled barcode reporters, as per the experimental design, was prepared following instructions on page 73 of the PhenoCycler-Fusion User Guide (PD-000011 REV M, Akoya Biosciences). Slides were prepared for the PhenoCycler-Fusion instrument according to the steps outlined in the PhenoImager-Fusion User Guide (PD-000001 Rev N, Akoya Biosciences). Briefly, the slide was moved from Storage Buffer to 1x PBS and incubated for 10 min. After incubation, a Flow Cell (Akoya Biosciences) was attached to the sample slide using the Flow Cell Assembly Device (Akoya Biosciences). The slide with the attached Flow Cell (Sample Flow Cell) was then placed in 1x PhenoCycler buffer for 10 min. PhenoCycler Fusion software (Version 2.2.0) was used to set up the imaging run on the PhenoCycler-Fusion, following the steps on page 57 of the PhenoImager-Fusion User Guide (PD-000001 Rev N, Akoya Biosciences).

Reagents were prepared and loaded into the appropriate reagent reservoirs on the instrument, and the pre-prepared reporter plate was loaded into the PhenoCycler. A new PhenoCycler run was initiated using the experimental protocol design. A blank flow cell was loaded into the Flow Cell Slide Carrier, and all software prompts during the pre-flight routine were followed. The Sample Flow Cell was then loaded into the carrier, and a leak check was performed. Scan regions were selected following automated sample finding, and imaging was started. Upon completion, the Sample Flow Cell was placed in the Storage Buffer at 4°C. The generated QPTIFF data file was used for downstream image analysis.

##### MERSCOPE Slide Preparation (STAMP-M)

Cells were STAMPed onto poly-D-Lysine coated MERSCOPE coverslips (Vizgen, 20400100) and stored at 4°C with desiccant until processing. Samples were rehydrated in 1× PBS and permeabilized overnight in 70% ethanol. STAMPs were then processed following the Formalin-Fixed Paraffin-Embedded Tissue Sample Preparation user guide (Vizgen, 91600112 Rev D), starting from page 28, step II.9. Briefly, STAMPs were decrosslinked at 90°C for 15 min using a decrosslinking buffer (Vizgen, 20300115), then washed twice with conditioning buffer (1 min each) followed by a 30-min incubation at 37°C. This was followed by a 2 h incubation in Anchoring Pretreatment at 37°C. STAMPs were then blocked with blocking solution for 1 h at room temperature and stained using the Cell Boundary Stain Kit (Vizgen, 10400118) for 1 h with primary stain solution. After washing with 1× PBS, samples were incubated with the secondary stain solution. Subsequently, STAMPs underwent sample prep washing (Vizgen, 20300001) and formamide wash buffer treatment (Vizgen, 20300002) for 30 min at 37°C. RNA anchoring was performed using anchoring buffer (Vizgen, 20300117) for 16 h at 37°C. STAMPs were then embedded in a thin polyacrylamide gel composed of Gel Embedding Premix (Vizgen, 20300118), Ammonium Persulfate (Millipore-Sigma, 09913-100G), and N,N,N’,N’-Tetramethylethylenediamine (TEMED) (Millipore-Sigma, T7024-25ML), followed by incubation at room temperature for 1.5 h. Samples were then cleared using a 1:100 proteinase K-containing clearing solution (NEB, P8107S) and Clearing Premix (Vizgen, 20300114) for 1 h at 37°C until transparent. Autofluorescence quenching was performed using the MERSCOPE Photobleacher (Vizgen, 10100003) for 4 h at room temperature. Probe hybridization was conducted with the PanCancer Pathways 500-gene Panel (Vizgen, 20300008) at 37°C for 48 h.

##### MERSCOPE Setup and Data Acquisition

Cells were STAMPed MERFISH measurements were carried out using the MERSCOPE platform, following the MERSCOPE User Guide (Vizgen, 91600001 Rev H). For data processing, RNA molecules were decoded using MERlin (Vizgen, software version v233). Cell segmentation was performed using Cell Boundary Stain signals and an on-board deep learning-based segmentation algorithm (Cellpose v1).

##### H&E Staining and Imaging

For post-Xenium (STAMP-X) Hematoxylin and Eosin (H&E) staining, slides were quenched in 10 mM sodium hydrosulfite (Sigma Aldrich, 157953-5G) at RT for 10 min, rinsed three times in water, and then immediately processed through the following sequence: once in water for 2 min, once in Mayer’s Hematoxylin (Sigma Aldrich, MHS16) for 20 min, three times in water for 1 min each, once in bluing solution (Dako, CS702) for 1 min, once in water for 1 min, once in 70% ethanol for 3 min, once in 95% ethanol for 3 min, once in Eosin Y Solution, Alcoholic (Leica, 3801615) for 2 min, twice in 95% ethanol for 30 seconds each, twice in 100% ethanol for 30 seconds each, and twice in xylene for 3 min each, as described in the Demonstrated Protocol Xenium HE Staining (CG000613 Rev B, 10x Genomics). The slides were dried for 15 min and then cover slipped using 1.5 mm thick cover glass and Cytoseal Mountant XYL (ProSciTech, 1A013-XYL-118) or toluene-free mounting media (Dako, CS705). For post-CosMx (STAMP-X-CP/CP/C) H&E staining, the glass flow cell coverslip was first removed by adhering clear sticky tape to the top of the flow cell, scoring around the inside of the adhesive edges of the flow cell, then peeling off the sticky tape, which left the adhesive edges attached to the slide and exposed the tissue for staining. Slides were then washed by dipping into water several times to remove any glass shards. The Demonstrated Protocol Xenium HE Staining (CG000613 RevB, 10x Genomics) was then followed starting from step 1.4, as described for post-Xenium H&E staining. Stained sections were covered using custom-cut coverslips fitted inside the flow cell adhesive edges using a glass scribe. Once the mounting media had dried, slides were scanned using a NanoZoomer 2.0HT (Hamamatsu) with a 40X objective or a Zeiss AxioObserver 7 with a 20x objective (Zeiss). For post-PhenoCycler (STAMP-X-PCF/PCF) H&E staining, the Sample Flow Cells were removed from the Storage Buffer and placed in a coplin jar containing xylene for 24 h. The Flow Cells were then carefully removed and disposed of properly. The slides were transferred to 100% ethanol for 2 min, dipping 10-15 times to ensure full coverage of the tissue. This step was repeated using 95% ethanol, followed by DI water. The slides were then placed in Mayer’s Hematoxylin for 4 min, followed by a rinse in DI water for 1 min, Bluing Reagent for 1 min, another DI water rinse for 1 min, and stained in Alcoholic Eosin for 2 min. Subsequent washes included 95% ethanol for 1 min, 100% ethanol for 1 min, fresh 100% ethanol again for 1 min, xylene I for 1 min, and finally, the slides were held in fresh xylene. Inside a fume hood, each slide was removed one at a time, the back was dried, and the slide was tilted onto a paper towel to remove excess xylene without allowing the tissues to dry. DPX or another xylene-based mountant was applied over the tissue area using a disposable Pasteur pipette while the remaining slides were kept in xylene to prevent over-drying. The glass coverslip was swiftly cleaned, any particles were removed, and the long edge was placed onto the slide. The slide was tipped towards the user, allowing the mountant to contact the coverslip, and was gently pressed down until the mountant spread evenly across the tissue. Excess mountant was carefully blotted using a paper towel, avoiding contact with the top of the coverslip. Any bubbles over the stained tissue were gently pressed out. The mountant was allowed to cure in the hood for at least 20 min before imaging. The slides with coverslips were then imaged using the NanoZoomer-SQ Digital slide scanner (Hamamatsu) with a 40x objective.

#### Chromium Fixed RNA Profiling of Cancer Cell Lines and hESC and Sequencing

MCF-7, LNCaP, SK-BR-3, iPSC, and hESC cell lines were processed using the Fixation of Cells & Nuclei for Chromium Fixed RNA Profiling protocol (CG000478, RevD, 10x Genomics). After quenching, the cells were counted in replicates using the Luna-FX7 Automated Cell Counter, and 0.5 to 1 million cells were subjected to either the Chromium Fixed RNA Kit (PN-1000474, 10x Genomics) for cancer cell lines or the Chromium Fixed RNA Kit (PN-1000475, 10x Genomics) for iPSC lineages and hESC BMP4 treatments. Single-plex or multiplex gene expression libraries were prepared according to the user guides CG000691 (RevB) and CG000527 (RevF), respectively. The libraries were quality controlled using a 5200 Fragment Analyzer System (Agilent, HS NGS Fragment Kit, DNF-474-1000) and sequenced on a NovaSeq 6000/X instrument following 10x Genomics’ user guide recommendations.

### DATA ANALYSIS

#### Fixed RNA Profiling (Flex)

The FASTQs files of the public 10x Genomics Flex dataset (downloaded from https://www.10xgenomics.com/datasets/320k_Human_PBMCs_Sub_Pool_16-plex_GEM-X_FLEX), used for benchmarking with STAMP, originally at 39,000 mean reads/cell have been downsampled to 10,000 reads per cell (as for 10x Genomics recommendations) with seqtk and a downsample ratio of 0.28. The Flex datasets were aligned to probe set reference from 10x Genomics using cellRanger v8.0 with the filter-probes argument set to false. Filtered feature-barcode matrices were loaded into *R* (v4.4.1) as a *SingleCellExperiment* object using the *read10XCounts* function from the *DropletUtils* package (v. 1.24.0).^50,51^

##### Quality Control

Quality control metrics were computed for each cell using the *addPerCellQC* function from the *scater*^52^ package (v1.32.0). Outliers in the number of counts and detected features were identified and removed using the *isOutlier* function from *scater* with parameters *type* = "*lower*", *log = TRUE*, and *nmads = 2* for counts and *nmads = 2.5* for detected features, respectively. Cells with a high percentage of mitochondrial gene expression were filtered out by applying *isOutlier* with *type* = "*higher*", log = *FALSE*, and *nmads = 5*.

##### Pre-processing

The count data were log-normalized using the *logNormCounts* function from *scater*. Feature selection was performed by modeling the mean-variance relationship with the *modelGeneVar* function from the *scran*^50^ package (v1.32.0). Highly variable genes were selected using *getTopHVGs* with an FDR threshold of 0.7. Principal Component Analysis (PCA) was conducted using the *fixedPCA* function from *scran*, specifying the *subset.row* argument to include the selected highly variable genes. The first 25 PCs were selected for downstream analyses based on the variance explained. UMAP dimensionality reduction was then applied using the *runUMAP* function from *scater* on these components.

##### Clustering

Cells were clustered using the *clusterCells* function from *scran*, specifying *dimred = "PCA"* and *BLUSPARAM = NNGraphParam(k = 50, cluster.fun = "louvain")*.

##### Doublet Identification

Potential doublets were identified using the *scDblFinder* function from the *scDblFinder* package^53^ (v1.16.0), incorporating cluster information via the *clusters = colLabels(sce)* argument, and subsequently removed by looking at the *scDblFinder.score* metrics together with canonical markers exclusive of specific populations.

#### CosMx RNA Datasets

##### Cell segmentation in AtoMx

Default cell segmentation yielded suboptimal results (e.g. over-segmentation and / or over-expanded cell boundaries) as assessed by visual inspection of the immunofluorescence images (e.g. DAPI, panCK, CD45 and CD298/B2M). Thus, we re-run cell segmentation for all STAMP-C experiments within the AtoMx platform using *Configuration C* (*Cell Pellet Array*) and setting the *Basic Parameters* as follows: *CellDilation* at 2 μm, *CellDiameter* at 30 μm for cell lines and 10 μm for PBMCs and *NuclearDiameter* at 10.8 μm for cell lines and at 5 μm for PBMCs. We additionally adjusted the *Advanced Parameters* for STAMP-C shown in Figure 1 and Figure S1 as follows: *BackgroundPercentile* at 0.4, *LogBlurSigma* at 2, *LoGThreshold* at 5, *Nuclei* and *CytoplasmModels* at CP, *NucleiProbability* at −2, *CellProbability* at −3, *CellFlowThreshold* at 0.1 and *MinCellSize* at 4.3. Flat-files exported from AtoMx were imported into R (v4.4.1), generating SingleCellExperiment objects using the SingleCellExperimens package^54^ (v1.26.0).

##### Quality Metrics

For each cell, we considered the cell area, number of counts and number of detected genes (the latter computed using the *addPerCellQC* function from the *scater* package (v1.32.0)). Lower and Higher outlier cells in any of these distributions were identified and removed using the *isOutlier* function from *scater* with the parameter *log=TRUE*. The *nmads* parameter, which specifies the number of median absolute deviations for outlier detection, was determined independently for each dataset based on visual inspection of the distributions. Additionally, cells located within 30 pixels of any field of view border were filtered out.

##### Clustering

Cells were clustered using the unsupervised approach provided by the *InSituType* (IST)^16^ package (v1.0.0), with the *n_clust* parameter varying according to the annotation step.

Background noise was calculated using the mean negative probe counts. The *fastCohorting* function was used to perform cohorting on mean immune fluorescence values (CD298/B2M, CD45, PanCK, DAPI, CD68_CK8_18), cell area, and aspect ratio. Cells with a posterior assignment probability below 0.8 were excluded from further analysis.

##### Marker genes

IST generates cluster profiles by aggregating counts for each gene within each cluster, and correcting for background (mean negative probe count). To compute marker genes, we normalized these cluster-level counts using the *normalizeCounts()* function from the *scater* package, and calculated the log_2_ fold change between each cluster and the average across remaining clusters (adding a small constant of 1e-6 to both).

##### UMAP

Prior to dimensionality reduction, the count matrices were normalized by total counts and log1p-transformed. Principal Component Analysis (PCA) was performed using the *prcomp_irlba* function from the *irlba* package^55^ (v2.3.5.1), a subset of PCs chosen based on the elbow plot, and Uniform Manifold Approximation and Projection (UMAP) was applied using the *umap* function from the *uwot* package^56^ (v0.2.2).

##### Signature Scoring

The circulating tumor cell mimics signature was derived by subsetting a 10x Genomics Flex dataset containing peripheral blood mononuclear cells (PBMCs) and MCF-7 cells, retaining only genes present in the CosMx 1K panel. We then applied the *scoreMarkers* function from the *scran* package (v1.32.0) to identify marker genes. The top 100 markers were then selected by highest absolute log2FC and the signature was subsequently scored in the PBMCs-CTCs CosMx dataset using the *AUCell_run* function from the *AUCell* package^18^ (v1.26.0).

##### Differentiation trajectory

After the preprocessing of the hESC samples, we used more strict thresholds to remove low quality cells. More specifically, for the STAMP-C samples we removed cells with fewer and more than 300 and 600 genes and 1500 and 6000 counts, respectively. For the Flex samples we removed cells with fewer and more than 2000 and 1000 genes and 5000 and 60000 counts, respectively. Then, for each time point, we normalized and scaled the data using the NormalizeData() and ScaleData() functions of Seurat (version 5.0.1) followed by a principal component analysis and clustering using 30 principal components (PCs) and a clustering resolution of 0.1. In the STAMP-C samples, as we expected the distinct cell states to be uniformly distributed across the FOVs, we decided to remove cell clusters that were present in less than half of the FOVs. We then inferred a differentiation trajectory using the Palantir python package (version 1.0.0). For each STAMP-C and Flex data, we first created a subset of ~40,000 cells and then computed an augmented affinity matrix using the 10 main PCs. Briefly, Palantir constructs this by augmenting the kNN graph affinity matrix with mutually nearest neighbors between successive time points. This matrix forms the basis to generate a force directed layout for visualization and as input for computing the diffusion operator which can be used for trajectory detection. We generated the force directed layout using 200 iterations. We then clustered the cells using the determine_cell_clusters() function of Palantir using the default parameters and 30 PCs. This generated 16 and 25 cell clusters for the STAMP-C and Flex data respectively, that were aggregated and annotated into nine cell types using canonical gene markers. To generate the Amnion, Endoderm and Mesoderm trajectories we determined the cell of origin and the terminal cells for each trajectory and ran the run_palantir() function with 500 waypoints. Gene expression trends of selected marker genes along the trajectories’ pseudotime were generated with the run_magic_imputation() and compute_gene_trends() functions.

##### Cell Communication Analysis

To evaluate the performance of STAMP in identifying key cell-cell communication, we analyzed the LPS condition at 4 hours using CellChat (v2.1.2),^57^ an R package designed to identify intercellular communication from single-cell RNA sequencing data. We normalized the gene expression data and assigned cell type labels. To infer intercellular communication, we leveraged CellChat’s curated ligand-receptor database^24^ and calculated communication probabilities using computeCommunProb(). These interactions were then aggregated into a comprehensive communication network with aggregateNet(). Key signaling pathways and their ligand-receptor pairs were identified through computeCommunProbPathway(), while netAnalysis_computeCentrality() determined the roles of different cell types as senders, receivers within the network. The signaling landscape was visualized using CellChat-implemented functions, including circle plots and heatmaps.

#### Xenium Datasets

##### Quality Metrics

Low-quality cells were filtered out by applying data-driven adaptive thresholds to the distributions of counts, genes, and cell area, following the same approach as with the CosMx datasets.

##### Pre-processing

SingleCellExperiment objects of the Xenium datasets were log-normalized using the l*ogNormCounts* function from the *scuttle* package^52^ (v1.14.0). For the Immune-Oncology datasets, no feature selection was performed. PCA was conducted using the *fixedPCA* function from *scran* with the parameter *BSPARAM=IrlbaParam()*. The proportion of variance explained by each principal component guided the selection of components for downstream analyses. UMAPs were then generated using the *runUMAP* function from the *scater* package.

##### Clustering

We constructed a shared nearest neighbors (SNN) graph using the *buildSNNGraph* function from *scran*, specifying *type="jaccard"*, k = 50, and *BNPARAM=AnnoyParam()*. Louvain clustering was performed on this graph using the *cluster_louvain* function from the *igraph* package^58,59^ with resolutions 0.5 or 1 depending on the annotation step. Following the initial round of annotation to identify major lineages, a second round of pre-processing and clustering was conducted within these lineages to achieve more detailed annotation.

##### Feature Selection

Following an initial annotation round – conducted by applying the aforementioned steps to the entire panel – and only in the 5K panel datasets, we performed feature selection to identify highly variable genes (HVGs) within PBMC lineage subsets. The mean-variance relationship was modeled using the *modelGeneVar* function from *scran*, and HVGs were selected using the *getTopHVGs* function setting the *fdr.threshold = 0.9*. PCA was then rerun as previously described, incorporating the *subset.row* parameter to specify the selected HVGs.

#### MERSCOPE Datasets

Cells presenting one of fewer than 20 transcripts per cell, 20 unique genes per cell, cell area > 800, cell area < 5 were classified as low-quality and excluded from the dataset. A Seurat object was constructed containing high quality cells and expression values were log-normalized using the NormalizeData() function from the package Seurat (v5.1.0). To identify gene markers for individual cell types, a one-vs-all differential expression analysis was performed using the FindAllMarkers() function in Seurat, genes with an adjusted p-value > 0.01 and avg_log2_FC < 0.5 were removed as false positives. The top 4 genes per cell line were selected based on the avg_log2_FC value and plotted as a dot plot for visualization.

#### STAMP Protein Datasets

##### CosMx Protein

Cell segmentation and initial pre-processing were performed using AtoMx. Flat files were downloaded, and background fluorescence was subtracted from each segmented cell to control for non-specific fluorescence signals. Cells with aggregated signals less than 20 or greater than 10,000, as well as those with an area smaller than 40 um^2^, were excluded from further analysis. Seurat was used for dimensionality reduction and clustering, selecting all 42 proteins for analysis. Data was normalized by applying centered log ratio transformation (CLR) across features. Principal component analysis was performed with nPCs set to 40, and after examining the elbow plot, the top 15 PCs were chosen for constructing the neighborhood graph (sNN, k.param = 20, distance = “euclidean”). Louvain clustering was then conducted with a resolution of 0.8. Cell annotation was achieved by inspecting the expression of each protein in the panel across the identified clusters, resulting in the classification of cells into eight main categories. For quality control of cells profiled in CosMx Protein after Xenium and those profiled in CosMx Protein alone, aggregated expression was calculated for each protein. Using the R packages ggpubr and ggplot2, a scatter plot was generated to compare the average expression of each protein across the different experiments. A linear regression line was added to represent the best-fitting line through the data points, along with Pearson correlation statistics to assess the relationship between the datasets.

##### Akoya PhenoCycler

Cell segmentation was performed using StarDist in QuPath (v0.5.0) with a custom Groovy script. The following parameters were used for the StarDist function: threshold = 0.5, channel = 0, normalizePercentiles = (1, 99), pixelSize = 0.5, cellExpansion = 5.0, and cellConstrainScale = 1.5. Fluorescence measurements were exported in text format. An expression matrix was constructed, retaining only the mean intensity measurements per channel for each segmented cell. Seurat (v5.1)^60^ was used for quality control and downstream analysis. Cells with a mean aggregated intensity below 200 or an area smaller than 20 um^2^ were excluded. Data normalization was performed using the centered-log-ratio option of the NormalizeData function in Seurat. All 40 proteins were included in the calculation of 40 principal components, and after reviewing the elbow plot, the top 15 PCs were selected for calculating the neighborhood graph. Louvain clustering was then applied with a resolution of 0.5 for unsupervised clustering.

#### External Datasets

##### 10x Genomics’ 5’ and 3’ Datasets

The 10x genomics 5’ and 3’ datasets containing healthy PBMCs were downloaded from https://www.10xgenomics.com/datasets/10k-human-pbmcs-5-v2-0-chromium-x-with-intronic-reads-2-standard and https://www.10xgenomics.com/datasets/10-k-pbm-cs-from-a-healthy-donor-v-3-chemistry-3-standard-3-0-0, respectively. The FASTQs files, originally sequenced at 24,782 and 54,286 mean reads/cell, were downsampled with seqtk with a downsampling ratio of 0.8 and 0.36, respectively. The datasets were aligned to the human reference genome GRCh38 from 10x Genomics using cellRanger v8.0 counts and multi for the 3’ and 5’ datasets, respectively. Filtered feature-barcode matrices were loaded into *R* (v4.4.1) as a *SingleCellExperiment* object using the *read10XCounts* function from the *DropletUtils* package and pre-processed as previously described for the RNA Flex datasets.

##### CITE-Seq Dataset

We obtained a publicly available PBMC CITE-Seq dataset from the GEO Series GSE164378, specifically selecting the samples labeled "PBMC CITE-seq ADT 3P." The raw data, consisting of measurements for 228 proteins, was normalized using the centered log-ratio (CLR) transformation, implemented via the NormalizeData function in Seurat. To facilitate comparison with the STAMP proteome datasets, we grouped the provided cell type annotations into broader immune lineages: B cells, T cells, Myeloid cells, and NK cells.

### QUANTIFICATION AND STATISTICAL ANALYSIS

Statistical analyses were performed in R 4.4.1. Pearson correlation values in Figures 2G, 2I, 3B, 3C, and 4G were calculated using the stat_cor() function from the ggpubr package^61^ (v0.6.0) with method = "pearson". Spearman correlation values in Figures S1A and S7F were calculated using the same function with method = "spearman". Volcano plots in Figure 4F and Figure S2J were generated using a pseudobulk analysis. First, counts were aggregated across group variables using the aggregateAcrossCells() function from the scuttle package (v1.16.0) with use.assay.type = "counts" and statistics = "sum". Next, a DESeq2^62^ object was created using the DESeqDataSetFromMatrix() function with design = ~ experiment, where experiment represents the replicates. Finally, DESeq was run, and results were visualized using the EnhancedVolcano() function from the EnhancedVolcano package^63^ (v1.24.0) with the following parameters: FCCutoff = 0.25, PCutoff = 0.05, and pCutoffCol = "padj".

## Supplementary Material

Supp 1

2

## Figures and Tables

**Table:** KEY RESOURCES TABLE

REAGENT or RESOURCE	SOURCE	IDENTIFIER
Antibodies		
Keratin 14	Akoya Biosciences	Cat# 4450031; RRID: AB_3095339
CD4	Akoya Biosciences	Cat# 4550112; RRID: AB_3094499
HLA-A	Akoya Biosciences	Cat# 4450046; RRID: AB_3678453
CD44	Akoya Biosciences	Cat# 4450041; RRID: AB_2936081
CD107a	Akoya Biosciences	Cat# 4550098; RRID: AB_3474468
CD20	Akoya Biosciences	Cat# 4450018; RRID: AB_2915939
beta-Actin	Akoya Biosciences	Cat#4450040; RRID: AB_3478101
CD68	Akoya Biosciences	Cat# 4550113; RRID: AB_2935894
CD66	Akoya Biosciences	Cat#4550001; RRID: AB_3475664
CD45RO	Akoya Biosciences	Cat# 4550127; RRID: AB_3662766
Pan-Cytokeratin	Akoya Biosciences	Cat# 4450020; RRID: AB_3083456
CD45	Akoya Biosciences	Cat# 4550121; RRID: AB_3674468
Vimentin	Akoya Biosciences	Cat# 4450050; RRID: AB_2935889
CD11c	Akoya Biosciences	Cat# 4550135; RRID: AB_3678450
CD8	Akoya Biosciences	Cat# 4250012; RRID: AB_2915960
IDO1	Akoya Biosciences	Cat# 4550123; RRID: AB_3476035
CD56	Akoya Biosciences	Cat# 4250087; RRID: AB_2936082
DC-LAMP	Akoya Biosciences	Cat# DDX0191P-100
FOXP3	Akoya Biosciences	Cat# 4550071; RRID: AB_2927679
HLA-DR	Akoya Biosciences	Cat# 4550118; RRID: AB_3080864
CD45RA	Biolegend	Cat# 304102; RRID: AB_314406
PCNA	Akoya Biosciences	Cat# 4550124; RRID: AB_2936083
CD14	Akoya Biosciences	Cat# 4450047; RRID: AB_3083457
Granzyme B	Akoya Biosciences	Cat# 4250055; RRID: AB_3472025
PD-L1	Akoya Biosciences	Cat# 4550072; RRID: AB_3096406
CD3e	Akoya Biosciences	Cat# 4550119; RRID: AB_2936080
PD-1	Akoya Biosciences	Cat# 4550038; RRID: AB_3096407
Ki67	Akoya Biosciences	Cat# 4250019; RRID: AB_2895046
CD15	Biolegend	Cat# 301902; RRID: AB_314194
ICOS	Akoya Biosciences	Cat# 4550117; RRID: AB_3096408
LAG3	Akoya Biosciences	Cat# 4550058; RRID: AB_3096409
Collagen I	Merck Millipore	Cat# AB758
Keratin 8/18	Akoya Biosciences	Cat# 4550082; RRID: AB_3676533
Estrogen Receptor	Akoya Biosciences	Cat# 4250074
CD38	Akoya Biosciences	Cat# 4250080; RRID: AB_3082976
CD79a	Akoya Biosciences	Cat# 4450078; RRID: AB_3082977
Epcam	Akoya Biosciences	Cat# 4450088; RRID: AB_3674473
p63	Akoya Biosciences	Cat# 4550081
beta-Catenin	Akoya Biosciences	Cat# 4250091; RRID: AB_3082978
Keratin 5	Akoya Biosciences	Cat# 4250090; RRID: AB_3676532
GATA4	Santa Cruz Biotechnology	Cat# sc-25310; RRID:AB_627667
Nestin	Sigma Aldrich	Cat# SAB4200347
PAX6	Atlas Antibodies	Cat# AMAB91372, RRID: AB_2716656
SOX2	Thermo Fisher Scientific	Cat#14-9811-82, RRID: AB_11219471
Oct3/4	Santa Cruz Biotechnology	Cat# sc-5279, RRID: AB_628051
TRA-1-60	Thermo Fisher Scientific	Cat# MA1-023, RRID: AB_2536699
Biological samples		
PBMCs	STEMCELL Technologies	200-0470
Human Peripheral Blood Leukopak, frozen	STEMCELL Technologies	200-0132
Chemicals, peptides, and recombinant proteins		
BSA	Miltenyi Biotec	130-091-376
Liberase TH	Roche	792347
growth factor-reduced Matrigel	Corning	354230
mTeSR+ growth media	STEMCELL Technologies	85850
DPBS	Sigma-Aldrich	D8537
Gentle Cell Dissociation Reagent	STEMCELL Technologies	100-0485
human recombinant BMP4	STEMCELL Technologies	78211
mFreSR	STEMCELL Technologies	05855
E8 media	ThermoFisher Scientific	A2656101
RPMI	ThermoFisher Scientific	12633012
RPMI (L-Glutamine)	ThermoFisher Scientific	11875093
B27	ThermoFisher Scientific	A1895601
BMP4	PeproTech	120-05ET-500UG
CHIR99021	STEMCELL Technologies	72052
IWR1	STEMCELL Technologies	72562
DMEM/F12	ThermoFisher Scientific	11320033
NEAA	ThermoFisher Scientific	11140050
SB431542	STEMCELL Technologies	100-1051
LDN193189	Sigma Aldrich	SML0559
Activin A	PeproTech	120-14E
FGF2	PeproTech	100-18B
Accutase	ThermoFisher Scientific	A1110501
DAPI	ThermoFisher Scientific	R37606
Formaldehyde	Sigma-Aldrich	252549-500ML
Cell Fixation and Permeabilization Buffer	10x Genomics	PN-2000517
Quenching Buffer	10x Genomics	PN-2000516
FBS	Thermo Fisher Scientific	16140071
Penicillin/Streptomycin	Gibco	15140122
DNase I	Worthington-Biochem	LS002007
LPS	InvivoGen	L2630-25MG
NBF stop buffer	Sigma	S6639-1L
Glycine	Sigma	G7126
NHS-acetate solution	ThermoFisher Scientific	26777
SSC	ThermoFisher Scientific	AM9763
formamide	ThermoFisher Scientific	AM9342
TE buffer	ThermoFisher Scientific	BP24731
CosMx Slide Preparation Kit, FFPE RNA	Nanostring a Bruker Company	121500006
DMSO	Sigma Aldrich	D8418
KCl	ThermoFisher Scientific	AM9640G
Tween-20	ThermoFisher Scientific	28320
Mayer’s Hematoxylin	Sigma Aldrich	MHS16
bluing solution	Dako	CS702
Eosin Y Solution, Alcoholic	Leica	3801615
Critical commercial assays		
Fixation of Cells & Nuclei for Chromium Fixed RNA Profiling protocol	10x Genomics	CG000478, RevD
CosMx Human Immuno-Oncology Protein Panel (64-plex assay)	Nanostring a Bruker Company	CMX-H-IOP-64P-P
Dynabeads^™^ Human T-Activator CD3/CD28 for T Cell Expansion and Activation	Thermo Fisher Scientific	11132D
Xenium Human Immuno-Oncology Panel	10x Genomics	PN-1000654
Xenium Prime 5k Human Pan Tissue and Pathways Panel	10x Genomics	PN-1000724
Xenium Prime 5k Mouse Pan Tissue and Pathways Panel	10x Genomics	PN-1000725
Xenium Multi-Tissue Stain Mix	10x Genomics	PN-2000991
Ribosomal RNA (18S rRNA)	10x Genomics	PN-2000991
Human RNA 6k Discovery Panel	Nanostring a Bruker Company	121500041
Human RNA Universal Cell Characterization	Nanostring a Bruker Company	CMX-H-USCP-1KP-R
Human RNA Universal Cell Segmentation	Nanostring a Bruker Company	121500020
Human RNA IO PanCK/CD45	Nanostring a Bruker Company	121500021
PanCancer Pathways 500-gene panel	Vizgen	20300008
Chromium Fixed RNA Kit	10x Genomics	PN-1000474
Deposited data		
Raw and analyzed data	This paper	GEO: GSE290468
Code	This paper	https://github.com/Single-Cell-Genomics-Group-CNAG-CRG/STAMP
Experimental models: Cell lines		
Breast cancer cell line	Dr. Alex Swarbrick (The Garvan Institute)	MCF-7
Breast cancer cell line	Dr. Alex Swarbrick (The Garvan Institute)	SK-BR-3
Prostate cancer cell line	Dr. Lisa Butler (South Australian Health and Medical Research Institute)	LNCaP
Reprogrammed fibroblasts	Polo Lab (Adelaide Centre for Epigenetics, University of Adelaide, Monash University)	iPSC 32F
Human embryonic stem cells	Sullivan Lab (Adelaide Centre for Epigenetics, University of Adelaide)	hESC
Human pancreatic beta cells	Dr. Lisa Nicholas (Adelaide Centre for Epigenetics, The University of Adelaide)	EndoC-betaH1
Human mammary epithelial cells (hTERT-immortalized)	Christopher Sweeney (South Australian immunoGENomics Cancer Institute, The University of Adelaide)	hTERT-HME1
Erythroleukemia	Dr. Winnie Kan (University of South Australia)	TF-1
Glioblastoma multiforme	Dr. Marina Kochetkova (University of South Australia)	U-373 MG
Tongue cancer-associated fibroblasts	Dr. Marina Kochetkova (University of South Australia)	Tongue Cancer CAFs
Head and neck squamous cell carcinoma	Dr. Marina Kochetkova (University of South Australia)	UMSCC-1 (Oral cancer cell line)
Epithelial (cancerous)	Dr. Greg Goodall (University of South Australia)	V16D
Neuroblastoma	Dr. Greg Goodall (University of South Australia)	CHP-134
Human embryonic kidney cells	Dr. Jason Gummow (Functional Genomics South Australia and Gene Silencing and Expression Laboratory, The University of Adelaide)	HEK293T
Neuroblastoma	Dr. Greg Goodall (University of South Australia)	SK-N-DZ
Neuroblastoma	Dr. Greg Goodall (University of South Australia)	SK-N-SH
Neuroblastoma	Dr. Greg Goodall (University of South Australia)	SHSY-5Y
Neuroblastoma	Dr. Greg Goodall (University of South Australia)	KELLY
Induced pluripotent stem cells	Polo Lab (Adelaide Centre for Epigenetics, University of Adelaide, Monash University)	MD3 iPSC
Induced trophoblast stem cells	Polo Lab (Adelaide Centre for Epigenetics, University of Adelaide, Monash University)	MD3 iTSC
Human dermal fibroblasts	Polo Lab (Adelaide Centre for Epigenetics, University of Adelaide, Monash University)	MD3 HDF
Bone marrow-derived hematopoietic stem cells	STEMCELL Technologies	70002
Prostate cancer FFPE block	Dr. Lisa Butler (South Australian Health and Medical Research Institute)	31944 1N
Prostate cancer FFPE block	Dr. Lisa Butler (South Australian Health and Medical Research Institute)	31568 F
Experimental models: Organisms/Strains		
Scavenger mice C57BL/6JArc. Source: Colony originally produced at the Animal Resources Centre (ARC) in Perth, Western Australia. In 2023, Ozgene assumed the operations of ARC, and the strain is now maintained and supplied under the designation C57BL/6JOzarc at the Ozgene Animal Resource Centre (Ozgene ARC) in Perth. Strain Details: This strain is derived from the original C57BL/6J line from The Jackson Laboratory and has been maintained by the ARC in Australia. (https://www.ozgene.com/c57bl-6jozarc/)	Robertson Lab (The Robinson Institute, The University of Adelaide)	N/A
Software and algorithms		
ImageJ	Schneider et al.^48^	https://imagej.nih.gov/ij/
Xenium Explorer software suite v3.1.0	10x Genomics	https://www.10xgenomics.com/support/software/xenium-explorer/
AtoMx^™^ Spatial Informatics Platform (v1.3.2)	Nanostring, a Bruker company	https://nanostring.com/products/atomx-spatial-informatics-platform/atomx-sip-overview/
PhenoCycler Experiment Designer v2.1.0	Akoya Biosciences	https://www.akoyabio.com/phenocycler/
PhenoCycler Fusion software v2.2.0	Akoya Biosciences	https://www.akoyabio.com/phenocycler/
R	R-project	https://www.r-project.org/
Napari	Napari	https://napari.org/stable/
Other		
Matrigel-coated chamber slides	ThermoFisher Scientific, Nunc-Labtek	171080
Superfrost Plus Micro Slides	VWR	48311-703
Xenium slides	10x Genomics	PN-3000941
10x Genomics’ gaskets from the single cell reagent kits	10x Genomics	PN- 370017
Punch pliers	Total Tools	9070220SB
micro-Slide 8-well cell culture chamber	ibidi	80841
12-well cell culture chamber	ibidi	81201
Silicone gasket for ProPlate^®^ microarray system	Grace Bio-Labs	246875
40 μm strainer	Cell Strainer	PN 43-10040-40
MERSCOPE coverslips	Vizgen	20400100

## Highlights

- STAMP enables scalable and flexible single-cell genomics by imaging
- Cost-efficient single-cell profiling of millions of cells without sequencing
- Multimodal single-cell analysis of RNA, protein, cell structure, and morphology
- Versatile application in high-throughput research and clinical settings
