# Supplementary material for: STAMP: Single-cell transcriptomics analysis and multimodal profiling through imaging
Source: Cell. Author manuscript; Available in PMC 2025 Oct 24. (PMC12551790; doi:10.1016/j.cell.2025.05.027)
Supplement: Supp 1 [file NIHMS2117886-supplement-Supp_1.pdf]

Cell, Volume *188*

Supplemental information

STAMP: Single-cell transcriptomics analysis and multimodal profiling through imaging

Emanuele Pitino, Anna Pascual-Reguant, Felipe Segato-Dezem, Kellie Wise, Irepan Salvador-Martinez, Helena Lucia Crowell, Maycon Marção, Max Ruiz, Elise Courtois, William F. Flynn, Santhosh Sivajothi, Emily Soja, Ginevra Caratù, German Atzin Mora-Roldan, B. Kate Dredge, Yutian Liu, Hannah Chasteen, Monika Mohenska, Juan C. Nieto, Raymond K.H. Yip, Ruvimbo D. Mishi, José M. Polo, Mohmed Abdalfttah, Adrienne E. Sullivan, Jasmine T. Plummer, Holger Heyn, and Luciano G. Martelotto

Cell line letter ID	Cell line name	Tissue origin	Culture conditions
A	PBMC	Peripheral Blood Mononuclear Cells (*STEMCELL Technologies, 200-0470*)	NA
B	MCF-7	Breast cancer	DMEM (*Gibco, 11965-092*) + 10% FBS (*Gibco, 16000-044*), 2 mM L-Glutamine (Gibco, 25030-081)
C	SK-BR-3	Breast cancer	McCoy (modified) 5A medium (*ThermoFisher Scientific, 16600082*),10% FBS (*Gibco, 16000-044*)
D	LNCaP	Prostate cancer	RPMI 1640 (*Gibco, 11875-093*), 10% FBS (*Gibco, 16000-044*)
E	iPSC 32F	Reprogrammed fibroblasts	See "Tri-lineage Differentiation of Induced Pluripotent Stem Cells" methods section
F	hESC	Human embryonic stem cells	See "Differentiation of Human Embryonic Stem Cell (hESC)" methods section
G	EndoC-betaH1	Human pancreatic beta cells	DMEM with 5.6 mM glucose (*Gibco, 11885-084*), 2% BSA fraction V (*Sigma-Aldrich, A9647*), 50 µM 2-mercaptoethanol (*Sigma-Aldrich, M6250*), 10 mM nicotinamide (*Sigma-Aldrich, N0636),* 5.5 µg/ml transferrin (*Sigma-Aldrich, T3309*), 6.7 ng/ml selenite (*Sigma-Aldrich, S5261*), 100 U/ml penicillin, and 100 µg/ml streptomycin (*Sigma-Aldrich, P4333*). On coated plates: DMEM 4.5g/L glucose *(Gibco, 11965-092),* Penicillin /Streptomycin 1% (*Sigma-Aldrich, P4333*). Fibronectin 2µg/ml (*Sigma-Aldrich, F0895*), ECM 1% (*Sigma-Aldrich, E1270*)
H	hTERT-HME1	Human mammary epithelial cells (hTERT-immortalized)	Mammary Epithelial Cell Growth Medium (MEGM) (*Lonza, CC-3150*), excluding the GA-1000 supplement
I	TF-1	Erythroleukemia	RPMI 1640, 2mM Glutamine (*Gibco, 11875-093*), 1% Sodium Pyruvate (*Gibco, 11360-070*), 2-5ng/ml Human GM-CSF (*R&D Systems, 215-GM-010/CF*), 10% FBS (*Gibco, 16000-044*)
J	U-373 MG	Glioblastoma multiforme	EMEM, 2mM L-Glutamine (*Sigma-Aldrich, M4655),* 1% Non-Essential Amino Acids (*ThermoFisher Scientific, 11140050*), 1mM Sodium Pyruvate (*Gibco, 11360-070*), 10% FBS (*Gibco, 16000-044)*
K	Tongue Cancer CAFs	Tongue cancer-associated fibroblasts	Primary Tongue SCC CAFs were purified from freshly excised tumours using enzymatic digestion, expanded ex vivo and cultured in DMEM *(Gibco, 11965-092),* 10% FBS (*Gibco, 16000-044*)
L	UMSCC-1 (Oral cancer cell line)	Head and neck squamous cell carcinoma	DMEM High Glucose (*Gibco, 11965-092*), 10% FBS (*Gibco, 16000-044*), Non-Essential Amino Acids (*ThermoFisher Scientific, 11140050*)

M	V16D	Epithelial (cancerous)	RPMI 1640 (*Gibco, 11875-093*), 10% FBS (*Gibco, 16000-044*)
N	CHP-134	Neuroblastoma	RPMI 1640 (*Gibco, 11875-093*), 10% FBS (*Gibco, 16000-044*)
O	HEK293T	Human embryonic kidney cells	DMEM (*Gibco, 10566-016*), 10% FBS (*Gibco, 16000-044*)
P	SK-N-DZ	Neuroblastoma	DMEM (*Gibco, 11965-092*), 10% FBS (*Gibco, 16000-044*)
Q	SK-N-SH	Neuroblastoma	DMEM (*Gibco, 11965-092*), 10% FBS (*Gibco, 16000-044*)
R	SHSY-5Y	Neuroblastoma	DMEM (*Gibco, 11965-092*), 10% FBS (*Gibco, 16000-044*)
S	KELLY	Neuroblastoma	RPMI 1640 (*Gibco, 11875-093*), 10% FBS (*Gibco, 16000-044*)
T	MD3 iPSC	Induced pluripotent stem cells	Essential 8™ Medium (*ThermoFisher Scientific, A1517001*), Matrigel® hESC-Qualified (*Corning, 354277*)
U	MD3 iTSC	Induced trophoblast stem cell	Okae media. DMEM/F-12, GlutaMAX (*ThermoFisher Scientific, 10565018*) with 0.3% (wt/vol) BSA, 0.2% (vol/vol) FBS (Cytiva, SH30084-03), 1% (vol/vol) ITS-X supplement (*ThermoFisher Scientific, 51500056*), 0.1 mM 2-mercaptoethanol (*ThermoFisher Scientific, 21985023*), 0.5% (vol/vol) pen–strep, 1.5 µg/ml l-ascorbic acid (*Sigma-Aldrich, A4544*), 5 µM Y27632 (*STEMCELL Technologies, 72304*), 2 µM CHIR99021 (*Miltenyi Biotec, 130-104-172*), 0.5 µM A83-01 (*Sigma-Aldrich, SML0788*), 1 µM SB431542 (*Tocris, S1067*), 50 ng/ml EGF (*Peprotech, AF-100-15*) and 0.8 mM VPA (*Sigma-Aldrich, P4543*) on Collagen IV treated plastics (*Sigma-Aldrich, C5533*)[1]
V	MD3 HDF	Human dermal fibroblasts	Opti-MEM (*ThermoFisher Scientific, 31985070*), FBS 10% (*Cytiva, SH30084-03*), Non-essential amino acids (*ThermoFisher Scientific, 11140050*)
W	BM CD34+	Bone marrow-derived hematopoietic stem cells (*STEMCELL Technologies, 70002*)	NA
X	31944 1N	Prostate tumour	2X 25 um scrolls from FFPE block, preparation of nuclei by snPATHO[2]
Y	31568 F	Prostate tumour	2X 25 um scrolls from FFPE block, preparation of nuclei by snPATHO[2]

Table S1. Human cell lines used in this study

1. Tan, J.P., Liu, X., and Polo, J.M. (2022). Establishment of human induced trophoblast stem cells via reprogramming of fibroblasts. Nat. Protoc. *17*, 2739–2759. https://doi.org/10.1038/s41596-022-00742-2.

2. Wang, T., Roach, M.J., Harvey, K., Morlanes, J.E., Kiedik, B., Al-Eryani, G., Greenwald, A., Kalavros, N., Dezem, F.S., Ma, Y., et al. (2024). snPATHO-seq, a

versatile FFPE single-nucleus RNA sequencing method to unlock pathology archives. Commun. Biol. *7*, 1–12. https://doi.org/10.1038/s42003-024-07043-2.

Target	Clone	PhenoCycler Barcode	PhenoCycler Reporter	Dilution (STAMP-PCF)	Dilution (STAMP-XPCF)
Keratin 14	Polyclonal	BX002	Atto 550	1:200	1:200
CD4	EPR6855	BX003	AlexaFluor 647	1:100	1:100
HLA-A	EP1395Y	BX004	AlexaFluor 750	1:200	1:200
CD44	156-3C11	BX005	Atto 550	1:400	1:400
CD107a	H4A3	BX006	AlexaFluor 647	1:200	1:400
CD20	L26	BX007	AlexaFluor 750	1:133	1:133
beta-Actin	W16197A	BX010	AlexaFluor 750	1:400	1:400
CD68	KP1	BX015	AlexaFluor 647	1:400	1:800
CD66	ASL-32	BX016	AlexaFluor 647	1:200	1:400
CD45RO	UCHL1	BX017	AlexaFluor 647	1:100	1:100
Pan-Cytokeratin	AE-1/AE-3	BX019	AlexaFluor 750	1:200	1:200
CD45	D9M81	BX021	AlexaFluor 647	1:200	1:133
Vimentin	091D3	BX022	AlexaFluor 750	1:400	1:400
CD11c	118/A5	BX024	AlexaFluor 647	1:200	1:200
CD8	C8/144B	BX026	Atto 550	1:200	1:200
IDO1	V1NC3IDO	BX027	AlexaFluor 647	1:200	1:200
CD56	CAL53	BX028	Atto 550	1:100	1:80
DC-LAMP	1010E1.01	BX030	AlexaFluor 647	1:200	1:133
FOXP3	236A/E7	BX031	AlexaFluor 647	1:100	1:100
HLA-DR	EPR3692	BX033	AlexaFluor 647	1:400	1:400
CD45RA	HI100	BX035	Atto 550	1:200	1:200
PCNA	PC10	BX036	AlexaFluor 647	1:400	1:800
CD14	EPR3653	BX037	Atto 550	1:200	1:400
Granzyme B	D6E9W	BX041	Atto 550	1:200	1:200
PD-L1	73-10	BX043	AlexaFluor 647	1:200	1:200
CD3e	EP449E	BX045	AlexaFluor 647	1:200	1:200
PD-1	D4W2J	BX046	AlexaFluor 647	1:200	1:133
Ki67	B56	BX047	Atto 550	1:200	1:200
CD15	HI98	BX050	AlexaFluor 647	1:400	1:800
ICOS	D1K2T	BX054	AlexaFluor 647	1:200	1:200
LAG3	EPR20261	BX055	AlexaFluor 647	1:200	1:200
Collagen I	Polyclonal	BX076	AlexaFluor 750	1:400	1:400
Keratin 8/18	C51	BX081	AlexaFluor 647	1:200	1:200

Estrogen Receptor	SP1	BX084	Atto 550	1:200	1:133
CD38	E7Z8C	BX089	Atto 550	1:200	1:100
CD79a	D1X5C	BX090	AlexaFluor 750	1:200	1:100
Epcam	D9S3P	BX091	AlexaFluor 750	1:200	1:200
p63	W15093A	BX093	AlexaFluor 647	1:200	1:200
beta-Catenin	12F7	BX096	Atto 550	1:400	1:200
Keratin 5	EP1601Y	BX101	Atto 550	1:200	1:200

Table S2. PhenoCycler Fusion run information.
