# Supplementary material for: STAMP: Single-cell transcriptomics analysis and multimodal profiling through imaging
Source: Cell. Author manuscript; Available in PMC 2025 Oct 24. (PMC12551790; doi:10.1016/j.cell.2025.05.027)
Supplement: 2 [file NIHMS2117886-supplement-2.pdf]

CellPress
OPEN ACCESS

Supplemental figures

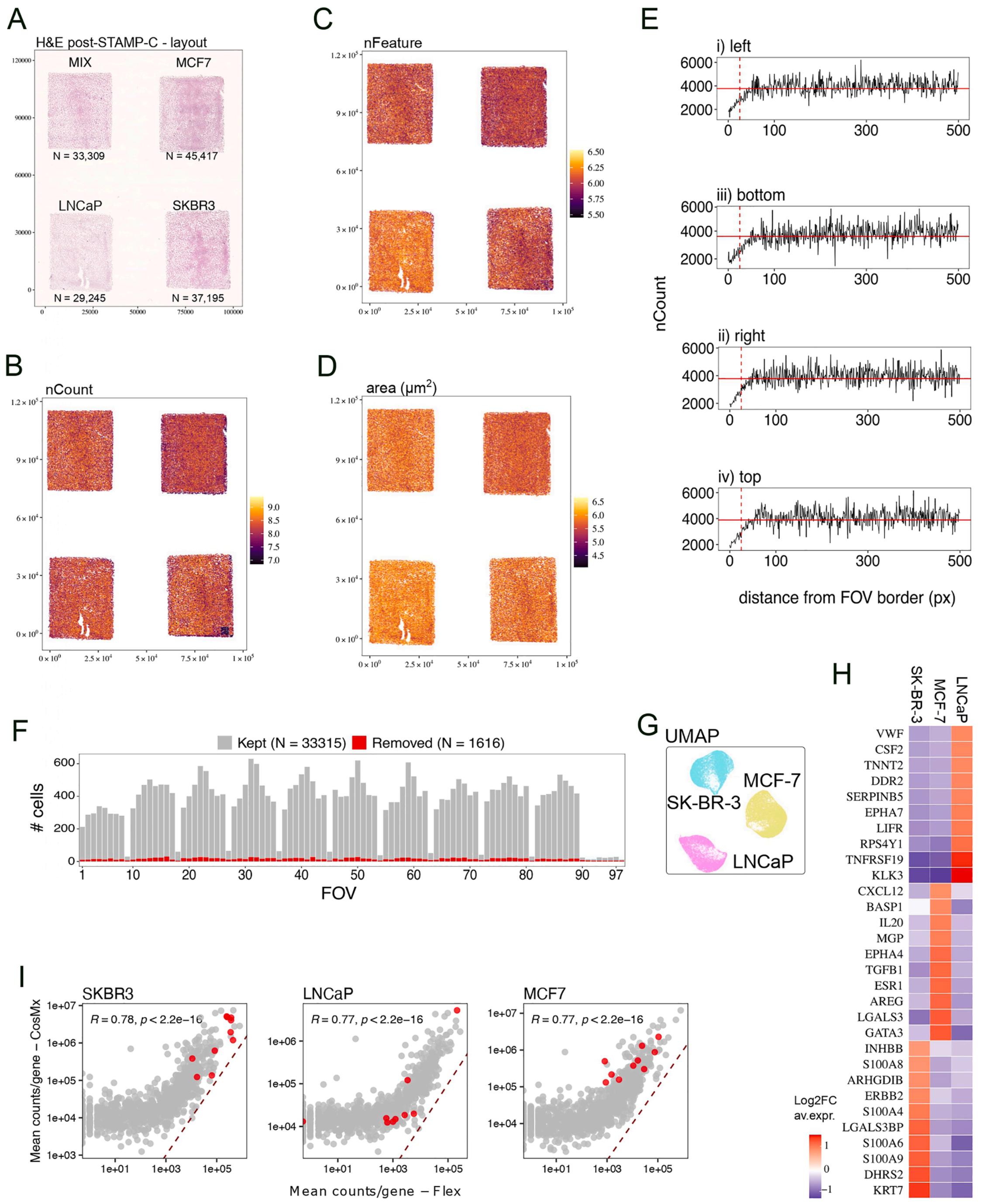

Figure S1. Clustering analysis of tumor cell lines in STAMP-C recapitulates classical single-cell transcriptomics data and can be combined with H&E, related to Figure 1

(A) H&E staining performed post-STAMP-C highlights single-cell and nuclear morphology while illustrating the experimental design.

(B–D) The spatial distribution of counts (B), features (C), and cell area (D) is depicted for each sub-STAMP.

(legend continued on next page)

(E) A line plot shows the total number of counts relative to the distance from the field of view (FOV) border, with horizontal red lines indicating the moving median and a vertical dashed red line marking the threshold applied.
(F) A bar plot quantifies the number of cells excluded from each FOV due to border effects.
(G) A UMAP visualization is color coded by cluster ID for MCF-7, LNCaP, and SK-BR-3 sub-STAMPs pooled together.
(H) A heatmap illustrates transcriptional profiles of each cell line as defined by InSituType (IST).
(I) A Spearman correlation plot compares gene expression between the Flex dataset and STAMP-C data from the same cell suspensions, with individual dots representing genes and red dots highlighting cell line marker genes identified in (F).

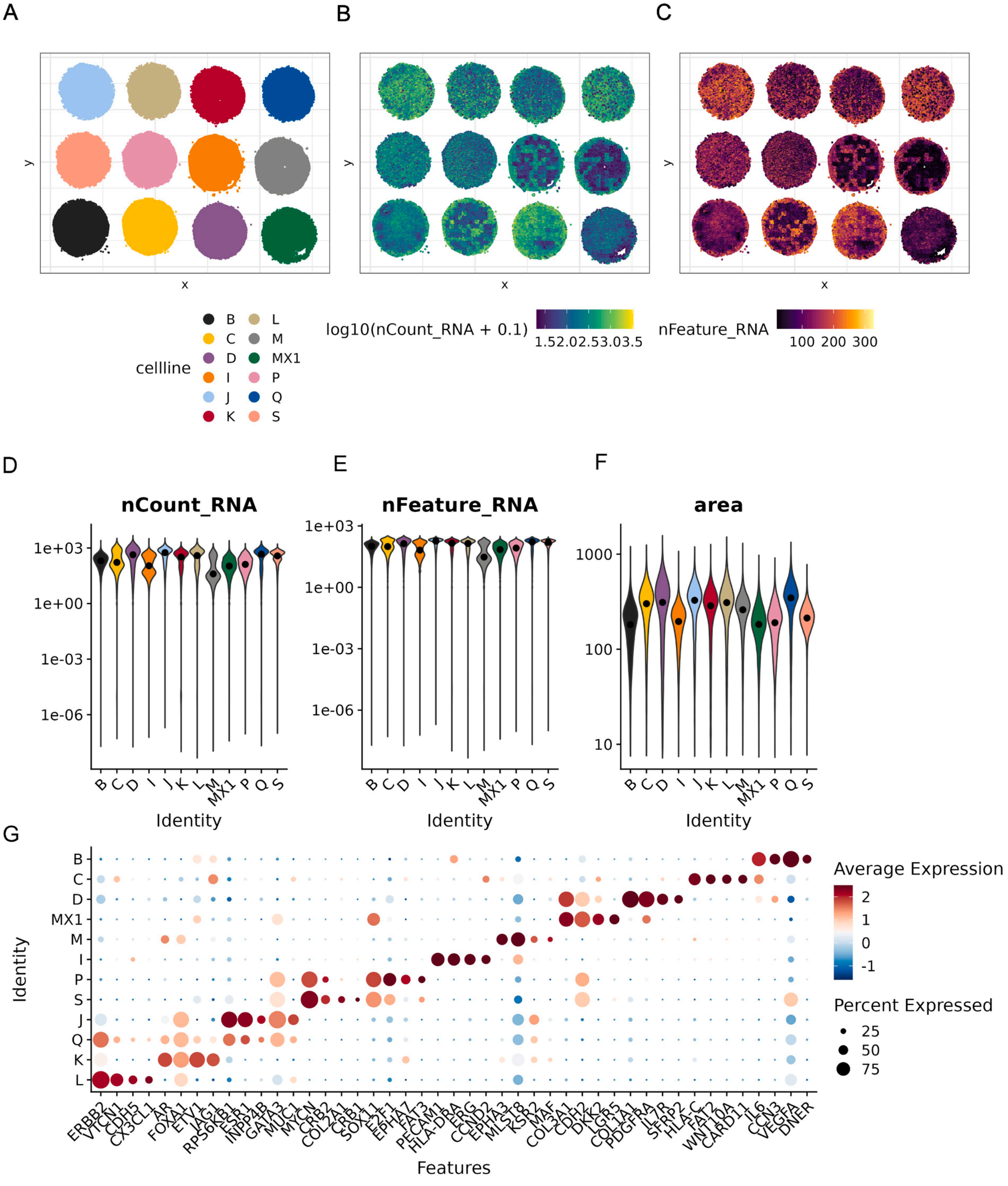

Figure S2. Assessment of STAMP using the MERSCOPE platform, related to STAR Methods

Quality control metrics and differential expression analysis results to identify key gene markers for 12 different cell lines profiled using the MERSCOPE platform.

(A) Layout of the STAMP-M experimental design colored by cell line (see Table S1 for cell line IDs and culture conditions).

(B) Spatial distribution of the number of transcripts per cell, in $\log_{10}$, for each cell line present.

(C) Spatial distribution of the number of unique genes captured per cell.

(D) Violin plot showing the distribution of the total transcript count per cell, in $\log_{10}$, for each cell line.

(E) Violin plot showing the distribution of the number of unique genes per cell, in $\log_{10}$, for each cell line.

(F) Violin plot showing the distribution of the cell area for each cell line.

(G) Dot plot showing the top 4 differentially expressed markers for each cell line.

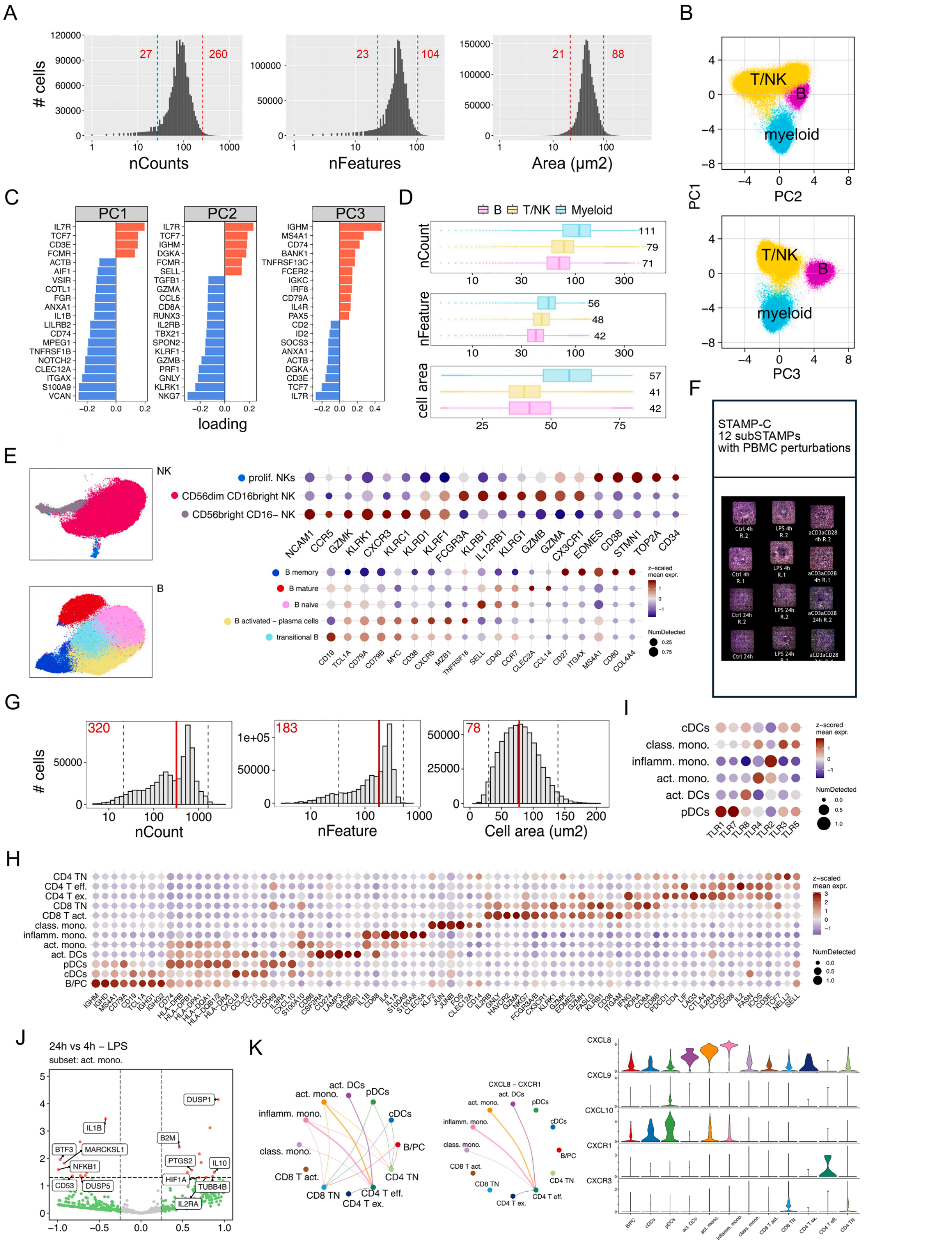

(legend on next page)

Figure S3. Profiling PBMCs with STAMP-X across platforms and panels, related to Figure 4

(A) Quality metrics of the 1.7M PBMC STAMP-X dataset showing the distribution of counts, features, and cell area before filtering. Red dotted lines and red text indicate the threshold set for filtering.

(B) Principal-component analysis (PCA) color coded by cluster identity, based on Leuven clustering of the STAMP-X PBMC dataset analyzed with the immuno-oncology panel.

(C) Loadings of the 3 first PCs.

(D) Boxplot display of the number of counts, features, and cell area split by cell lineage. Text labels indicate median values.

(E) UMAPs and dot plots of NK and B cell lineages showing population markers.

(F) PBMCs perturbations STAMP-C layout.

(G) Quality metrics distributions for (F). Red lines show median values, while dotted gray lines show filtering thresholds.

(H) Dot plot of annotated PBMCs populations with marker genes.

(I) Dot plot of myeloid sub-populations showing Toll-like receptors (TLRs) Z_{scored} mean expression.

(J) Volcano plot of differentially expressed genes in activated monocytes, comparing 24 h versus 4 h under LPS stimulation.

(K) CellChat cell-cell communication networks inferred from expression of ligand-receptor pairs. Circle plots showcasing the strength of ligand-receptor interactions (left) and strength of communication between CXCL8 and CXCR1 for any two cell types (middle). Thickness of line indicates higher number/communication probability. Violin plots showing the normalized gene expression for the chemokines and chemokine receptors detected (CXCL pathway) in the dataset by CellChat.

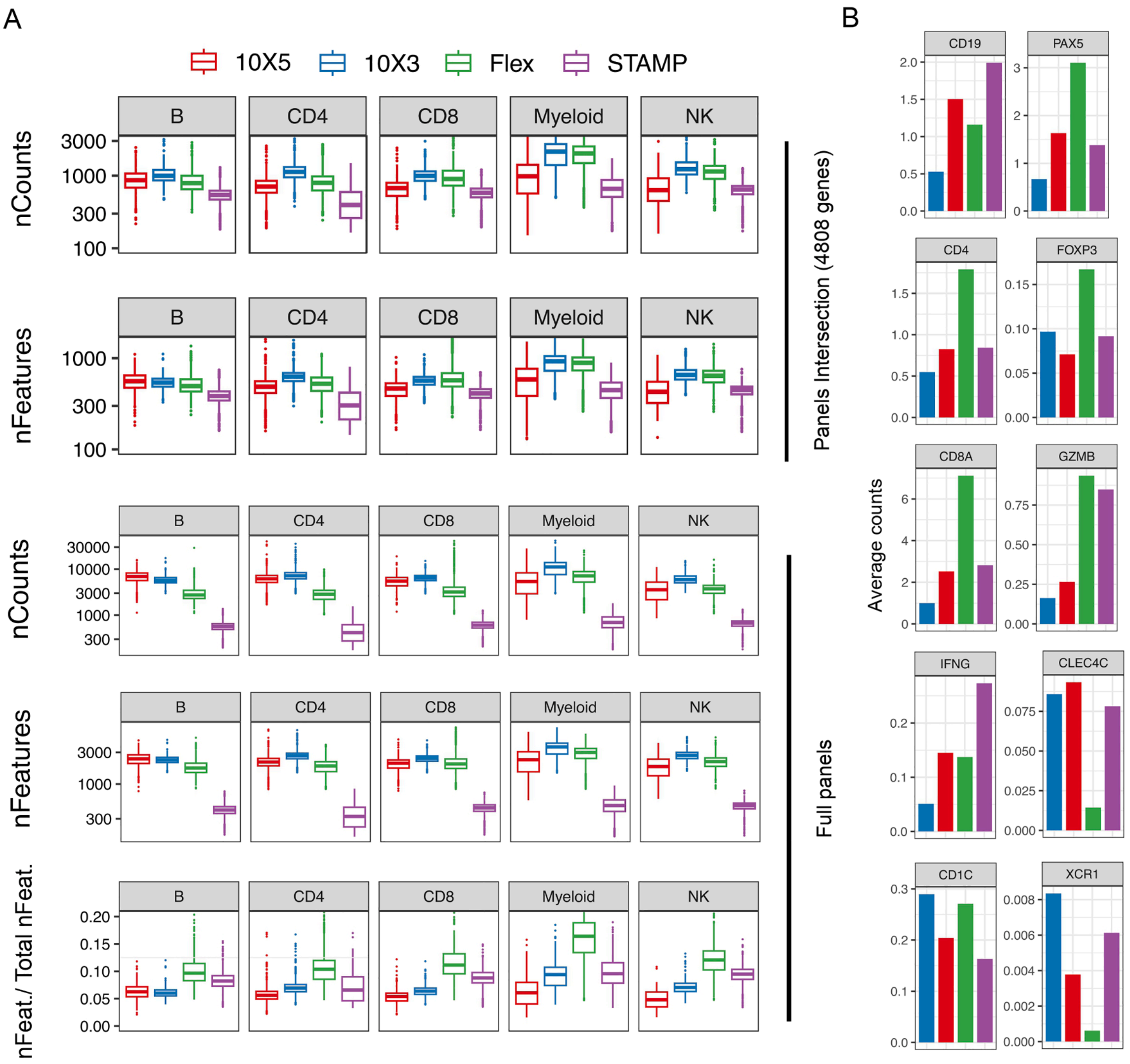

Figure S4. Benchmarking STAMP against classical single-cell analysis assays, related to Figure 4

Comparison of quality metrics between STAMP-X using the Xenium Prime 5k Human Pan Tissue and Pathways panel and 10× Genomics 3′, 5′, and Flex gene expression assays. All datasets have been downsampled to 7,000 cells.

(A) Boxplots showing the distribution and median of the number of counts and features for each immune cell population (full panels), also computed on the intersection between panels (panels intersection), and the percentage of recovered features over the total number of genes for each technology.

(B) Average counts of lineage marker genes calculated in B lymphocytes (CD19 and PAX5), CD4 lymphocytes (CD4 and FOXP3), CD8 lymphocytes (CD8A, GZMB, and IFNG), and myeloid cells (CD1C, CLEC4C, and XCR1).

CellPress
OPEN ACCESS

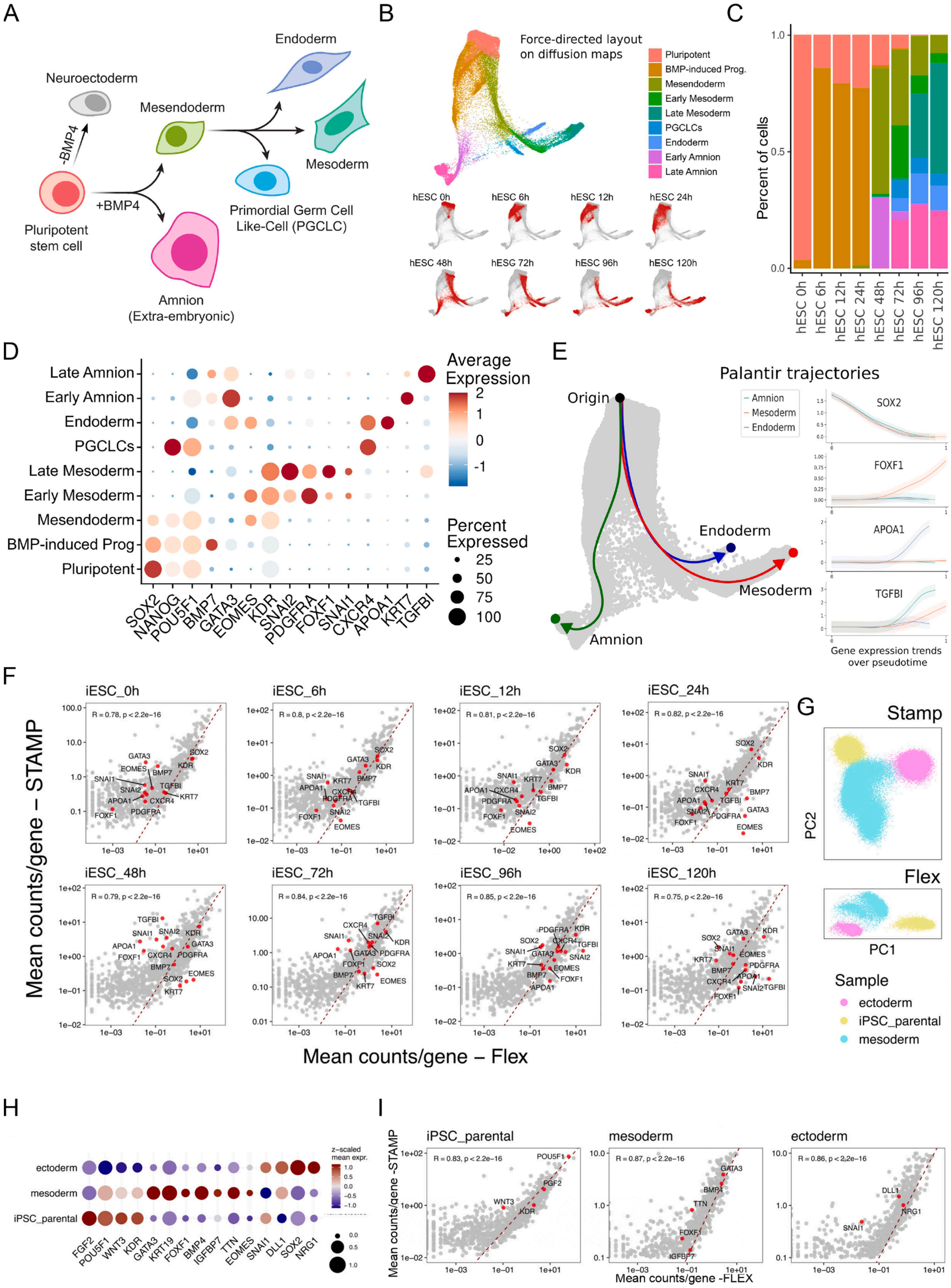

(legend on next page)

Figure S5. STAMP profiling of cell state dynamics during stem cell differentiation recapitulates classical single-cell transcriptomics data, related to Figure 5

Profiling human embryonic stem cell (hESC) differentiation upon BMP4 treatment with Single-Cell Gene Expression Flex technology.

(A) A schematic illustrates the anticipated cell trajectories following BMP4 treatment of hESCs.

(B) Cells from eight time points post BMP4 treatment are visualized using a force-directed layout on diffusion maps computed with the Palantir algorithm. The upper panel shows cells colored by their annotated states, while the lower panel separates cells by time point.

(C) A bar plot quantifies the proportion of each annotated cell state at each time point, using the same color scheme as in panel (B).

(D) A dot plot displays the key marker genes used to define each cell state.

(E) Differentiation trajectories for amnion, Endoderm, and mesoderm lineages were inferred with Palantir (right). Corresponding expression trends of selected marker genes are plotted along the trajectories' pseudotime (left).

(F) Gene expression correlation between STAMP and RNA Flex in hESCs at paired time points. R indicates the Pearson correlation, each dot is a gene, and red dots indicate key lineage-defining markers at each developmental stage, as shown in (D). Analysis of induced pluripotent stem cell differentiation (iPSC).

(G) Principal-component analysis (PCA) plot displaying PC1 and PC2, with points color coded by sub-STAMP identity to highlight clustering patterns.

(H) A dot plot showcases the key marker genes utilized for cell type annotation, indicating their expression levels across identified sub-STAMPs.

(I) Spearman gene expression correlation comparing the Flex dataset with the STAMP-C dataset derived from the same cell suspensions. Each dot represents a gene, while red dots highlight marker genes identified in (G) and (H).

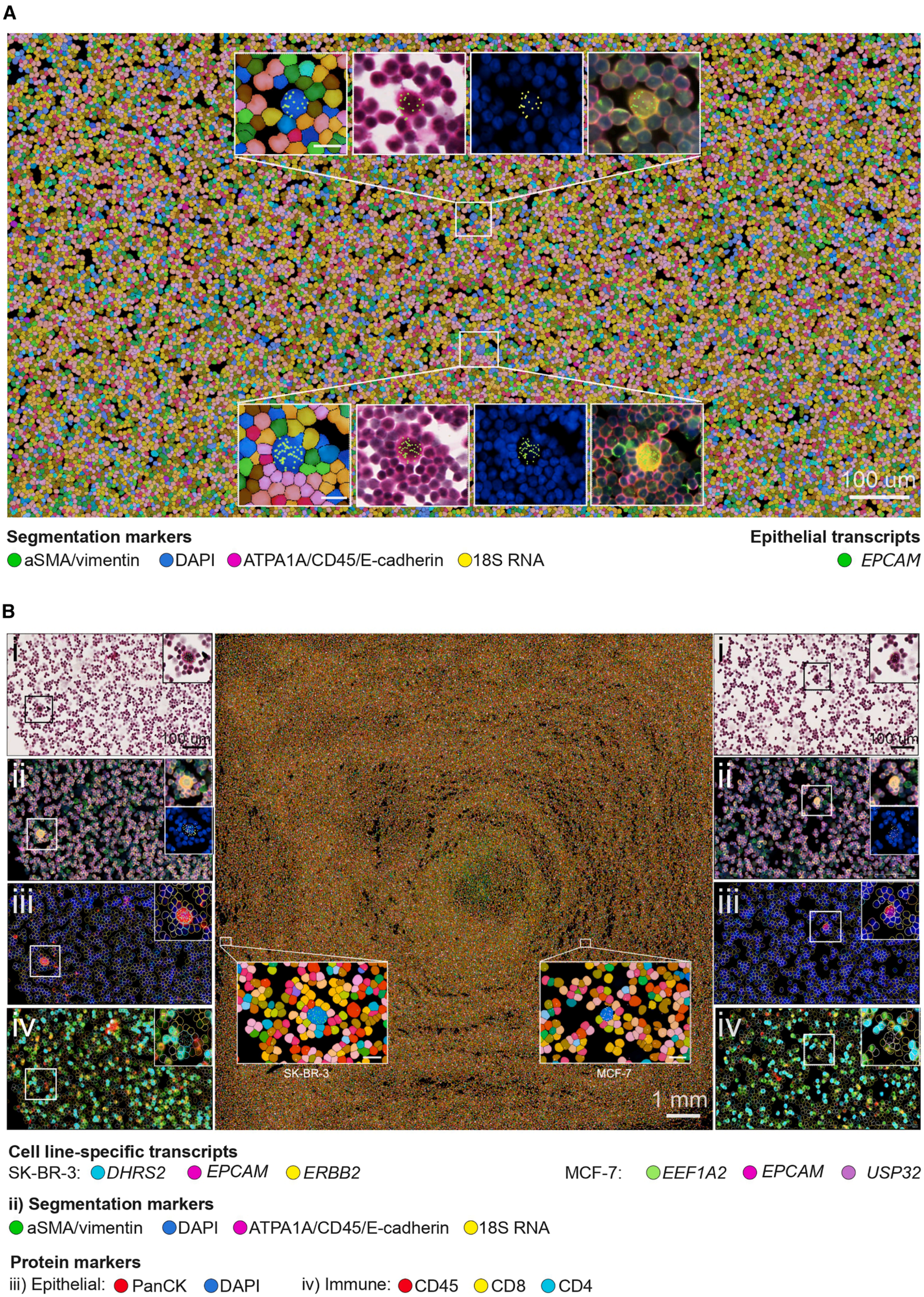

(legend on next page)

Figure S6. Identification of CTCs-mimic with STAMP-X and multimodal STAMP-X-CP, related to Figure 6

(A) Full STAMP-X showing cells clustered in Xenium explorer. Two regions of interest (ROIs) are shown as white frames, from where the zoom-in images are taken. Enlargements show cells color coded by cluster identity, hematoxylin and eosin staining (H&E), DAPI nuclear segmentation marker in blue, cytoplasmic and membrane segmentation markers ATP1A1/CD45/e-cadherin in pink, 18S RNA in yellow, and alphaSMA/Vimentin in green, in that order. *EPCAM* single transcripts for epithelial/cancer cells are shown as green dots in each image.

(B) Full STAMP-X-CP showing clustered cells with two ROIs (white frames), from where the zoom-in images are taken, with one SK-BR-3 and one MCF-7 cell. Enlargements show H&E staining (i), the fluorescence image with the segmentation markers (ii; DAPI nuclear segmentation marker in blue, cytoplasmic and membrane segmentation markers ATP1A1/CD45/e-cadherin in pink, 18S RNA in yellow, alphaSMA/Vimentin in green), and the protein markers used to identify epithelial/tumor cells (iii; DAPI nuclear staining in blue and PanCK in red) and to identify immune cells (iv; CD45 in red, CD8 in yellow, and CD4 in cyan). SK-BR-3-specific transcripts are shown as dots in the first column (*DHRS2* in cyan, *EPCAM* in magenta, and *ERBB2* in yellow) and MCF-7-specific transcripts are shown in the last column (EEF*1A2* in green, *EPCAM* in magenta, and *USP32* in purple).

CellPress
OPEN ACCESS

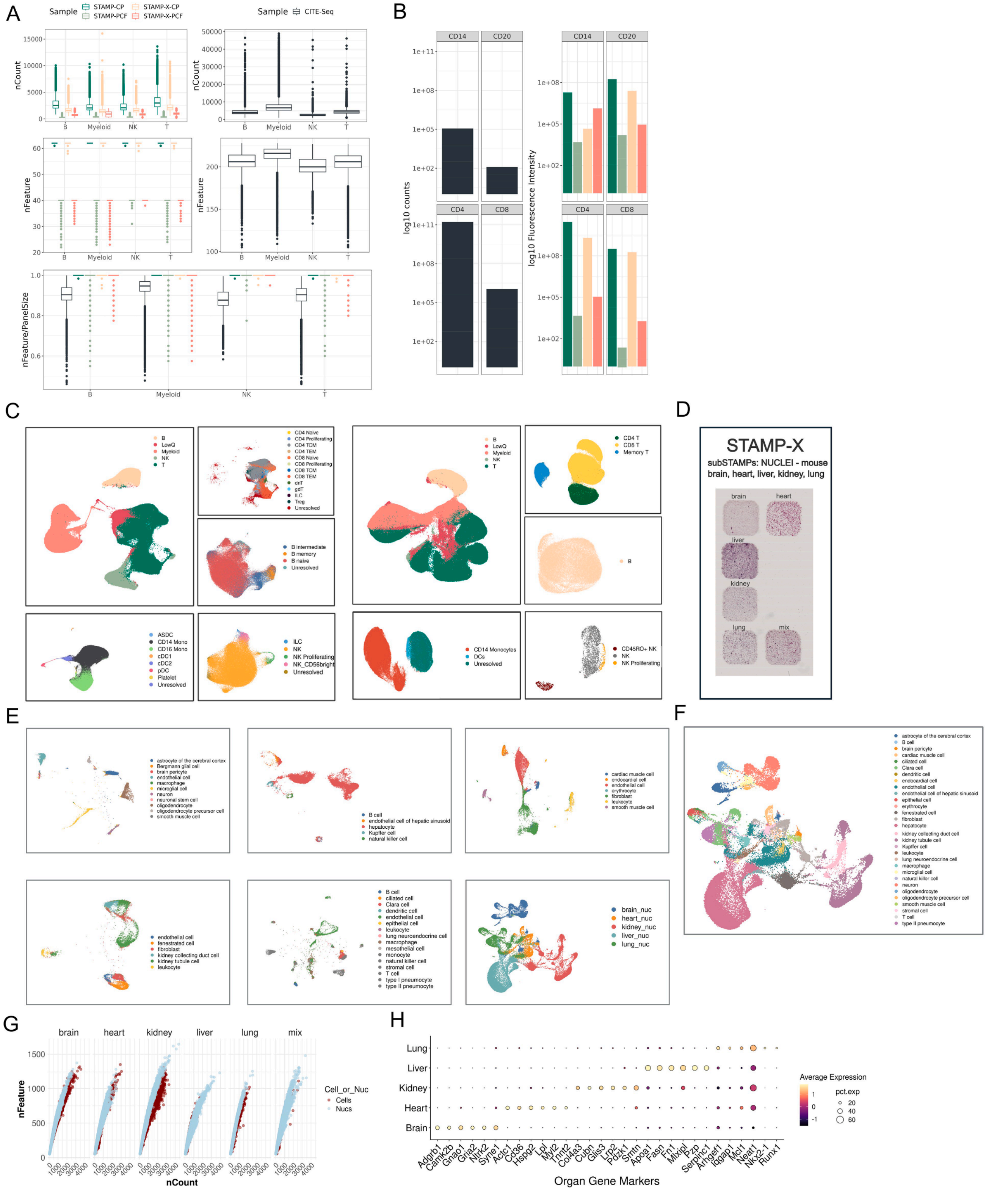

Figure S7. STAMP multimodal analysis of human PBMCs and of nuclei isolated from mouse organs, related to STAR Methods

(A) Boxplots showing the distribution of mean fluorescence intensities (MFI), the number of features (proteins), and the number of features normalized by the panel size in STAMP-CP, STAMP-X-CP, STAMP-PCF, and STAMP-X-PCF, as well as the number of counts in CITE-Seq (for each major cell population of PBMCs).

(legend continued on next page)

(B) Bar plots of the average counts and fluorescence intensities for key protein markers.
(C) UMAP visualization of RNA STAMP-X-PCF showing unsupervised clustering and cell type annotations, demonstrating that all clusters of PBMCs are detected with further sub-clustering of each major immune cell type (left). UMAP visualization of protein STAMP-X-PCF illustrates that all clusters of PBMCs are detected, with sub-clusters of each major immune cell type (right).
(D) STAMP layout of hematoxylin and eosin (H&E) staining of each subSTAMP performed on nuclei dissociated from mouse tissues: brain, heart, kidney, liver, and lung, as well as a 1:1:1:1:1 mixture of nuclei from these organs.
(E) UMAP visualizations of nuclei clusters from individual mouse tissues: (i) brain, (ii) heart, (iii) lung, (iv) liver, (v) kidney, and (vi) a mixed nuclei population from all five organs.
(F) UMAP visualization of all cell types identified in the mixed population from the five organs.
(G) Scatterplot of tissue STAMP transcript count by number of genes detected across five mouse tissue types for cells (burgundy dot) and nuclei (light blue dots).
(H) Dot plot highlighting the most highly expressed marker genes for each organ type.
